# Genomic Prediction of Breeding Values Using a Subset of SNPs Identified by Three Machine Learning Methods

**DOI:** 10.3389/fgene.2018.00237

**Published:** 2018-07-04

**Authors:** Bo Li, Nanxi Zhang, You-Gan Wang, Andrew W. George, Antonio Reverter, Yutao Li

**Affiliations:** ^1^CSIRO Agriculture and Food, St Lucia, QLD, Australia; ^2^Shandong Technology and Business University, School of Computer Science and Technology, YanTai, China; ^3^Shandong Co-Innovation Centre of Future Intelligent Computing, YanTai, China; ^4^Centre for Applications in Natural Resource Mathematics, University of Queensland, St Lucia, QLD, Australia; ^5^School of Mathematical Sciences, Queensland University of Technology, Brisbane, QLD, Australia; ^6^CSIRO Data61, Dutton Park, QLD, Australia

**Keywords:** machine learning methods, single nucleotide polymorphisms, genomic prediction, breeding values, beef cattle, live weight

## Abstract

The analysis of large genomic data is hampered by issues such as a small number of observations and a large number of predictive variables (commonly known as “large P small N”), high dimensionality or highly correlated data structures. Machine learning methods are renowned for dealing with these problems. To date machine learning methods have been applied in Genome-Wide Association Studies for identification of candidate genes, epistasis detection, gene network pathway analyses and genomic prediction of phenotypic values. However, the utility of two machine learning methods, Gradient Boosting Machine (GBM) and Extreme Gradient Boosting Method (XgBoost), in identifying a subset of SNP makers for genomic prediction of breeding values has never been explored before. In this study, using 38,082 SNP markers and body weight phenotypes from 2,093 Brahman cattle (1,097 bulls as a discovery population and 996 cows as a validation population), we examined the efficiency of three machine learning methods, namely Random Forests (RF), GBM and XgBoost, in (a) the identification of top 400, 1,000, and 3,000 ranked SNPs; (b) using the subsets of SNPs to construct genomic relationship matrices (GRMs) for the estimation of genomic breeding values (GEBVs). For comparison purposes, we also calculated the GEBVs from (1) 400, 1,000, and 3,000 SNPs that were randomly selected and evenly spaced across the genome, and (2) from all the SNPs. We found that RF and especially GBM are efficient methods in identifying a subset of SNPs with direct links to candidate genes affecting the growth trait. In comparison to the estimate of prediction accuracy of GEBVs from using all SNPs (0.43), the 3,000 top SNPs identified by RF (0.42) and GBM (0.46) had similar values to those of the whole SNP panel. The performance of the subsets of SNPs from RF and GBM was substantially better than that of evenly spaced subsets across the genome (0.18–0.29). Of the three methods, RF and GBM consistently outperformed the XgBoost in genomic prediction accuracy.

## Introduction

High-throughput genomic technologies have created enormous challenges to researchers with issues such as a small number of observations and a large number of predictor variables (commonly known as “*large P small N*” problem), high dimensionality or highly correlated SNP data structures (Chen and Ishwaran, 2012; González-Recio et al., 2014). Conventional statistical methods focusing on univariate hypothesis and assuming independent explanatory variables suffer significantly due to lack of power and accuracy for dealing with the complexity of multiple interactions or correlations among predictors (e.g., SNP-SNP and SNP-covariate interactions) (Lettre et al., 2007; Zheng et al., 2007; So and Sham, 2011; Adams et al., 2015).

Numerous statistical methods have been developed for improving predictability of large datasets with the “large P small N” problems, including parametric models – such as subset selection (Breiman, 1995; Fan and Li, 2001), LASSO (least absolute shrinkage and selection operator, Tibshirani, 1996), and SCAD (smoothly clipped absolute deviation penalty, Fan and Li, 2001), but they are all computationally demanding. Although LASSO and SCAD can be solved efficiently, they are regression-based with strong parametric assumptions and ignore dependence among explanatory variables. In recent years, non-parametric machine learning methods have been proved to be efficient in addressing these problems (Chen and Ishwaran, 2012; González-Recio et al., 2014). They do not require any prior knowledge on underlying genetic models (i.e., additive, dominance or recessive), and are excellent “black-box” approaches for pre-screening important predicting variables. Most importantly, they can detect SNP-SNP or SNP-covariate interactions (Lubke et al., 2013).

Since Meuwissen et al. (2001) pioneered the genome wide selection method using high-density SNP markers in breeding value prediction, there have been a number of studies that examined the influence of parametric and nonparametric methods on the predictability of phenotypic values (e.g., de los Campos et al., 2013; Howard et al., 2014; Okser et al., 2014; Jacquin et al., 2016; Waldmann, 2016). Using simulated SNP data with additive or two-way epistatic interactions, Howard et al. (2014) evaluated the prediction accuracy and mean squared error (MSE) of phenotypic values of 10 parametric and four nonparametric methods. These 10 parametric methods included least squares regression, ridge regression, Bayesian ridge regression, least absolute shrinkage and selection operator (LASSO), Bayesian LASSO, best linear unbiased prediction (BLUP), Bayes A, Bayes B, Bayes C, and Bayes Cπ. The four non-parametric methods included Nadaraya-Watson estimator, reproducing kernel Hibert space (RKHS), support vector machine (SVM) regression and neural networks. While they found that both genetic architecture and the heritability of the traits had great impacts on the estimates of accuracy and MSE (Howard et al., 2014), the non-parametric methods performed better than the parametric methods when the underlying genetic architecture was entirely due to epistasis. Recently, using both simulation data and real pig data, Waldmann (2016) also confirmed that in the presence of dominance and epistasis, the non-parametric machine learning method—BART (Bayesian additive regression trees, Chipman et al., 2010) gave a smaller genomic prediction error and increased prediction accuracy of phenotypic values than Random Forests, BLASSO, GBLUP and RKHS regression methods.

Among machine learning methods, the most popular method is Random Forests (RF, Breiman, 2001). It is a tree-based ensemble method for classification or regression of multiple variables (Chen and Ishwaran, 2012; Alarcon et al., 2015; Li et al., 2016). The method has been used in genetic association studies (Brieuc et al., 2015; Everson et al., 2015; Petralia et al., 2015; Stephan et al., 2015), epistasis detection for cancer identification and treatment (Pashaei, 2015; Shi and He, 2016), gene network pathway analysis (Pang et al., 2006; Wang et al., 2010; Chen and Ishwaran, 2012), prediction of protein DNA-binding sites from amino acid sequences (Wu et al., 2009) and protein-protein interaction sites in sequence (Sikic et al., 2009).

Another tree-based ensemble method, similar to RF but with a great improvement in the prediction error, is Gradient Boosting Machine (GBM) (Friedman, 2001, 2002; Schapire, 2003; Hastie et al., 2009). Walters et al. (2012) developed a sub-setting algorithm that deals with SNP linkage disequilibrium issue in GWAS when using RF and GBM, and found that the integrated approach provided a satisfying improvement in RF results. Lubke et al. (2013) showed that GBM was an efficient method in filtering SNPs and reducing complex models in multivariate phenotype GWAS analyses, but they did not go further to evaluate the efficiency of GBM in genomic prediction of breeding values. Using a trait from a simulated dataset, Ogutu et al. (2011) compared the prediction accuracy of genomic breeding values (GEBVs) for the trait from three machine learning methods (RF, GBM and SVM) and found that GBM performed the best, followed by SVM and then RF. However, they did not evaluate the efficiency of these methods in a real dataset, nor in selecting a subset of SNPs for genomic prediction.

Recently Chen and He (2015) introduced a new machine learning method - Extreme Gradient Boosting (XgBoost). It is based on the similar principle as GBM, but applies a more regularized model than GBM to control over-fitting. XgBoost runs at least 10 times faster than GBM (Fan and Xu, 2014; Chen and Guestrin, 2016). The method has been shown to outperform RF in some problem domains involving difficult learning tasks (e.g., dynamic music emotion recognition, Fan and Xu, 2014). Zhou and Troyanskaya (2015) applied XgBoost and a few other deep-learning based sequence models, and identified the functional effects of noncoding variants from re-sequencing data.

Genomic selection (GS) has revolutionized genetic improvement in dairy cattle (Hayes et al., 2009; Garrick, 2011; Boichard et al., 2016), poultry (WolC, 2014) and crop species (Crossa et al., 2017) thanks to its unparalleled ability to predict breeding values of animals and plants even without phenotypes. However, this benefit of the technology has not been fully realized in a number of animal species (e.g., meat and dairy sheep, Raoul et al., 2017; most of aquaculture species, Wang et al., 2017). The main reasons contributing to a slow adaptation of the technology in selective breeding programs include: 1) non-existence of commercially available large SNP panels due to the lack of quality reference genome sequences (Xiang, 2015); (2) the lack of breeding programs in which GS can be implemented (Xiang, 2015) and (3) the high cost associated with the need to genotype large numbers of individuals in reference populations for genomic prediction of target populations. Although rapid development of high-throughput technologies, commercial costs of genotyping a high density SNP panel per individual animal has been reducing at a fast speed, developing cost-effective methods for applying low-density SNP panels to build breeding populations for genomic selection still has profound impacts on many industries. In addition, a large number of SNPs in a high-density SNP panel that were used for genomic prediction of future phenotypes of animals had been shown to have very small or no effects on phenotypes (e.g., MacLeod et al., 2016). This really raises the question of whether there is merit in using only a small subset of SNPs that have direct relevance to biological functions of a trait of interest for genomic prediction of breeding values.

There have been a number of publications that applied machine learning methods for high dimension reduction of SNP datasets for GWAS (Liang and Kelemen, 2008; Walters et al., 2012; Lubke et al., 2013) and the genomic prediction of phenotypic traits (Long et al., 2011; Bermingham et al., 2015). Despite the reported advantages of GBM and XgBoost over RF, there has been no information available on the application of GBM and XgBoost in livestock genomic prediction. More specifically, the utility of these methods in identifying a subset of SNPs for genomic prediction of breeding values has not been examined before. The objective of this study was to evaluate the efficiency of three tree-based ensemble methods (RF, GBM and XgBoost) in the identification of a subset of SNPs and using them for genomic prediction of breeding values.

## Materials and methods

### Beef cattle datasets

Animal Care and Use Committee approval was not obtained for this study because historical data was used and no animals were handled as part of the study. Analysis was performed on phenotypic data and DNA samples that had been collected previously as part of the Australian Cooperative Research Centre for Beef Genetic Technologies (Beef CRC; http://www.beefcrc.com/). A SNP dataset consisting of 40,184 SNP markers from 2,093 tropical Brahman cattle was used for the study. The animals consisted of 1,097 Brahman bulls (called the “bull population”) and 996 Brahman cows (referred to as “cow population”). The bull population varying from 373 to 509 days old, came from 57 contemporary groups (defined as the combinations of location, herd and birth year) and were measured for live weight (the average weight being 308.64 kg (± 38.85 kg) with the range from 180 to 430 kg, Barwick et al., 2009). The cow population varying from 323 to 400 days old had a live weight ranging from 115 to 299 kg (average 209.75 kg). A quality check of 40,184 SNP markers resulted in the removal of 2,102 SNPs having MAF <0.01 or with missing genotypes due to the full genotype requirement by RF. A total of 38,082 SNPs with a 100% call rate was used for the final analysis. In this study, the bull population was used as a training dataset and the cow population as an independent validation population.

Unlike a mixed animal model that can accommodate fixed effects in the model, the machine learning methods are non-parametric approaches and cannot directly account for any environmental effects. Therefore, prior to any analysis, a linear model, in which the response variable was the live weight and the fixed effects were the contemporary group and age, was used to correct for environmental and age effects in the bull population. The new adjusted phenotypes after removing the significant fixed effects were then combined with the SNP data of the population for RF, GBM and XgBoost analyses. All analyses were performed using the R program (version 3.4.4, R Core Team, 2013).

### Supervised learning methods–RF, GBM, and XgBoost

All three machine methods RF, GBM, and XgBoost are supervised learning methods in which a training dataset with large number of predictors (e.g., SNPs, *X*_*i*_, where *X* refers to a vector containing genotypes of all SNPs for *i*^*th*^ animal) is used to predict a target phenotype (*y*_*i*_). The prediction value is a continuous variable. The fundamental part of a supervised learning method is about how to make the prediction *y*_*i*_ given *X*_*i*_. Normally it involves the identification of an objective function and optimizing it. The objective function usually comprises two parts—training loss function and regularization term (Friedman, 2002). The training loss function indicates how well a model fits on a training dataset (normally presented as a mean squared error MSE), while the regularization term measures the complexity of the model. In general, the more complicated a model becomes, the more unstable the results will be. Therefore, it requires a bias-variance trade-off between the two important components of an objective function.

The details of RF can be found in Breiman (2001). It comprises four main parameters: N – total number of observations, M – total number of predictor variables (SNPs), mtry – randomly chosen subset of M for determining a decision tree, normally mtry << M, and Ntree – total number of decision trees that form a forest. Briefly, the RF procedure is as follows-: (1) randomly select a subset of observations (by default two-third from all animals); (2) randomly select a subset of SNP markers – mtry (by default the squared root of M); (3) create a single tree by recursively splitting the subset of SNPs in the subset of the samples to form tree nodes, with the aim to separate the subset observation samples into two distinctive groups; During the splitting of a node in a tree, the SNP with the greatest ability to decrease the MSE of the child nodes is selected to split the node; (4) use all “out-of-bag” data (OOB, i.e., the remaining one-third animals) to determine the prediction MSE of the tree; For each variable (SNP) in the tree (model), then conduct random permutation of the SNP order in the tree and calculate the difference between new tree MSE and the initial MSE; (5) generate a forest of trees by repeating steps 1–4; (6) obtain final SNP variable importance values (denoted as VIM) by averaging prediction error values across all the trees in the forest containing that SNP. The process of node splitting continues until there is no more change of MSE values in all terminal nodes. For regression, a SNP VIM value is measured as %IncMse, which is the percentage of increased MSE after a SNP is randomly permuted in a new sample (Nicodemus and Malley, 2009; Nicodemus et al., 2010a,b). In RF, all SNPs are ranked based on their VIM values. These VIM values range from negative to positive values. A large positive value indicates a large increase in the prediction error (MSE) when the SNP is randomly permuted, in comparison to the MSE value prior to permutation, hence the more important the SNP is. On the other hand, negative values indicate that when these SNPs were randomly permutated, the prediction models from new SNP orders had a smaller prediction error than prior to permutation. In other words, these SNPs would be problematic if they were used for regression analysis of the live weight phenotype.

GBM builds a predictive model through an iterative way of assembling “weak learners” together (those regression decision trees with very small number of splits), then optimizes it using a cross validation method (Hastie et al., 2009). During the process, new models are added sequentially to minimize the prediction error made by a previous model until no further improvements can be made. At each split, a SNP is only chosen to split animal observations into two daughter nodes if the SNP can best increase the homogeneity in the daughter nodes (Lubke et al., 2013). The fundamental difference between RF and GBM is that RF applies the bootstrapping method to generate random samples from all observations with replacement as training datasets, and uses “out-of-bag” (OOB) samples as validation datasets. The final prediction of a SNP VIM value in RF is based on the average of the prediction errors of the SNP from all OOB datasets. While in GBM, multiple random samples from all observations are also chosen as training datasets, but these samples are not independent. Subsequent samples heavily rely on the weights of previous samples.

There are four important parameters that need to be predetermined in a GBM analysis aiming to select an optimal number of trees that can minimize the validation error. These include the number of trees (Ntree), learning rate (*shr*, determining a step scale in a gradient direction for overall prediction), maximum tree depth (determining the level of complex interactions between predictors, normally 1–10) and minimum samples per leaf. For regression, a SNP VIM value GBM produces is the relative influence. It is a maximal estimated improvement in MSE over a constant fit over all iterative trees (Friedman, 2001). In other words, it is the sum of decreased MSE values across all individual split points of all the trees generated by the boosting algorithm. Therefore, the larger the relative influence value is, the more important a SNP will be.

The algorithm of XgBoost is very similar to GBM, but much faster than GBM, since it can employ parallel computation (GBM is unable to do this). Most importantly, XgBoost can improve prediction errors by applying a more regularized model formalization to control over-fitting problems (Chen and He, 2015; Chen and Guestrin, 2016). In a supervised machine learning method, a regularization term of an objective function normally involves adding a penalty term to the loss function, a norm of weights vector that contains the learned parameters in the loss function. It penalizes large values of the weights in the loss function and therefore controls the overfitting problem of the loss function. The regulation term is always dependent on the loss function. However, in XgBoost, the second-order Taylor series is added to the original loss function used in the GBM method (mean squared error for regression). The regulation term is independent to the loss function, therefore, it simplifies and speeds the process of solving the optimal weights of leaf nodes in the tree.

There are a large number of parameters (a total of 18) that need to be predetermined in XgBoost, including 3 general, 12 booster and 3 task parameters. A SNP VIM value that XgBoost produces is the “Gain” value (*Gain*_*k*_ denotes the decrease in the prediction error of the objective function to split a node in a tree with the *k*^*th*^ SNP). The larger the value, the more important the SNP is. The detailed description of fundamental differences between XgBoost and GBM algorithms are given in the guide for XgBoost (Chen, 2014).

### Pre-determination of minimal parameter values required for RF, GBM, and XgBoost analyses using the bull population

Two crucial parameters impacting the outcome of a RF analysis include the size of forest trees (Ntree) and the number of markers at each sampling event (mtry) to form a tree. To determine the minimum requirement for these parameters, we systematically examined the impacts of a range of Ntree and mtry values on the average population MSE value of all SNPs using the bull population. These included Ntree = 500, 1,000, 1,500, 2,000, 2,500, … 5,000 (i.e., interval = 500), and mtry = 1, sqrt(*M*), 2^*^sqrt(*M*), or 0.1^*^M, where *M* is the total number of SNPs (38,082). The minimum values of the parameters were determined when the average MSE value of all SNPs reached a stable status in which increasing Ntree and other parameter values no longer changed the average MSE value. Then these parameters were used for the subsequent analyses. The R program library randomForest (Liaw and Wiener, 2002) was used.

For GBM and XgBoost, we applied the R libraries gbm (Ridgeway with contributions from sothers, 2017) and xgboost (Chen et al., 2017). The default values were chosen for the majority of the parameters other than Ntree and the learning rate (a step size shrinkage for avoiding variable overfitting) *shr* (for GBM) and *eta* (for XgBoost) values, in which we examined a range of values for Ntree = 500, 1,000, 1,500, 2,000, 2,500, …, 5,000 (i.e., interval = 500) and the learning rate *shr* for GBM or *eta* for XgBoost = 0.01, 0.04, 0.07, or 0.1 respectively. Again, we used the error rate curve to determine the minimum parameters required. The minimum parameters were reached when the average population MSE value reached a consistent status. That is, the value where increasing input parameters did not change the MSE trend.

### Genome-wide screening for top ranking SNPs with three methods using the bull population

Once the minimal values for the parameters - Ntree, mtry, *shr* (a shrinkage, also called learning rate for GBM), or *eta* (a step size shrinkage for XgBoost) were determined, they were used for the final run of individual machine learning methods. Based on the SNP VIM values from RF (%IncMSE), GBM (relative importance) and XgBoost (Gain), all SNPs were ranked from the most important to the least important ones. The top 400, 1,000, 3,000 SNPs as well as all SNPs with the positive VIM values were then identified from each method. These values are chosen largely due to the fact that in practice commercial companies for DNA genotyping are always carried out using the multiplexes of 96 or 384 wells.

### Gene ontology (GO) enrichment analysis

To see whether a subset of SNPs identified by each method has any biological relevance, we performed the GO analysis on the gene sets that are close to top ranking 1,000, 3,000 or all SNPs (using 10 kb as a distance limit for the closest gene) with the positive VIM values, using the program PANTHER (protein annotation through evolutionary relationship, Mi et al., 2013). The basic parameters applied included *Bos Taurus* (for organism), statistical overrepresentation test (analysis method), PANTHER GO-Slim biological process (annotation data set) and Fisher's Exact with FDR multiple test correction (test type). In addition, we also applied the UCSC's liftOver tool (minMatch = 0.1) (Hinrichs et al., 2006) to translate the bovine SNP genomic positions to human coordinates (GRC37/19) and used the GREAT program (v3.0.0, McLean et al., 2010).- GREAT assigns each gene a regulatory domain, default 5 kb upstream, 1 kb downstream plus distal up to 1,000 kb or until the nearest gene's basal domain, which associates with the gene GO term. As a consequence, GREAT performs a GO enrichment analysis at the gene-level using the hypergeometric test as well as a regulatory domain test based on binomial test, where it accounts for variability in gene regulatory domain size by measuring the total fraction of the genome annotated for any given ontology term and counting how many input genomic regions fall into those areas.

### Estimate of additive genetic variance using a genomic relationship matrix (GRM) constructed from the subset of SNPs of the cow population

Once the top-ranking SNPs were chosen with three machine learning methods using the bull population, the utility of these SNPs in predicting the additive genomic breeding values (GEBVs) of individual animals was validated with the cow population. To quantify the effects of the top 400, 1,000 and 3,000 SNPs on the live weight phenotype, we applied a linear mixed genomic model to estimate the genetic variance explained by each subset of selected SNPs. The model is as follows:

$$\begin{array}{l} \text{y}=1_{\text{n}}\mu +\text{X}\text{b}+\text{Z}\text{a}+\text{e} \end{array}$$

where μ is the population mean, **X**_(n__x__b)_ refers to a design matrix, **b**_(b__x__1)_ represents a vector of fixed effects consisting of contemporary groups and age, and n is the number of animals. **Z**_(n__x__n)_ is an incidence matrix, **a**_(n__x__1)_ refers to a vector of random SNP additive effects, and **e**_(__n__x__1)_ is a vector of errors. In the model, we assume the random effects **a** and **e** follow a normal distribution, with mean zero and variance $\sigma_{\text{a}}^{2}$*GRM* (where *GRM* is a genomic relationship matrix with its values calculated from the subset of SNP information) and **I**_(n__x__n)_ $\sigma_{\text{e}}^{2}$, respectively. Here, $\sigma_{\text{a}}^{2}$ and $\sigma_{\text{e}}^{2}$ are additive genetic and error variances. For GRM calculation, we used the same approach as VanRaden (2008). That is $GRM=\frac{WW^{t}}{2\overset{m}{\underset{k=1}{\sum }}Pjk\left(1-Pjk\right)}$, where *m* refers to the number of SNPs, W_(n__x__m)_ is a matrix containing all additive contributions from m SNPs of n animals. For a given j^th^ animal at *k*^th^ SNP locus, the additive contribution W_jk_ of the SNP with three genotypes AA, AB, and BB, is calculated as *2-2p*_*jk*_, *1-2p*_*jk*_, and *-2p*_*jk*_, respectively. Here, *p*_*jk*_ is the allele frequencies for allele B. The software Remlf90 (Misztal et al., 2002) was used to estimate variance components and to obtain GEBV from a model with a GRM from top 400, 1,000 and 3,000 SNPs, evenly spaced SNPs and all SNPs separately.

### Distribution of diagonal elements of GRMs constructed from subsets of SNPs

The quality of genomic data has an impact on the accuracy of genomic predictions. Simeone et al. (2011) suggested that the diagonal elements of a genomic relationship matrix (GRM) could be used for identifying secondary populations or mislabelled animals if multiple peaks were evident. Therefore, prior to the validation, we examined the distributions of the diagonal and off-diagonal elements of all GRMs constructed using the subsets of SNPs from the Brahman cow population.

### Five-fold cross-validation for determining accuracy of GEBVs using a subset of SNP markers

A five-fold cross-validation scheme was used to determine the accuracy of genomic prediction of a selected subset of SNPs in the cow population. The animals (996) were randomly split into 5 equal-size groups and each group with about 199 animals (20% of the population) was in turn assigned with missing phenotypic values and used as the validation set. The accuracy of genomic prediction was calculated as the correlation between the predicted GEBVs of the animals with no phenotypic values and the corrected phenotypes of the animals, divided by a square root of the heritability value. The corrected phenotypes were derived after adjusting the original phenotypes for the fixed effects of contemporary group and age (i.e., = phenotype–fixed effects). The accuracy reported in the study was the average of the accuracies of genomic prediction from 5-fold groups.

For comparison purposes, we also calculated the accuracies of genomic prediction from all the SNPs (38,083), the SNPs with positive VIM values from each machine learning method, as well as 400, 1,000, and 3,000 SNPs that were selected to be evenly spaced across the genome.

## Results

### Minimal parameter determination for individual machine learning methods

**Figure 1:**
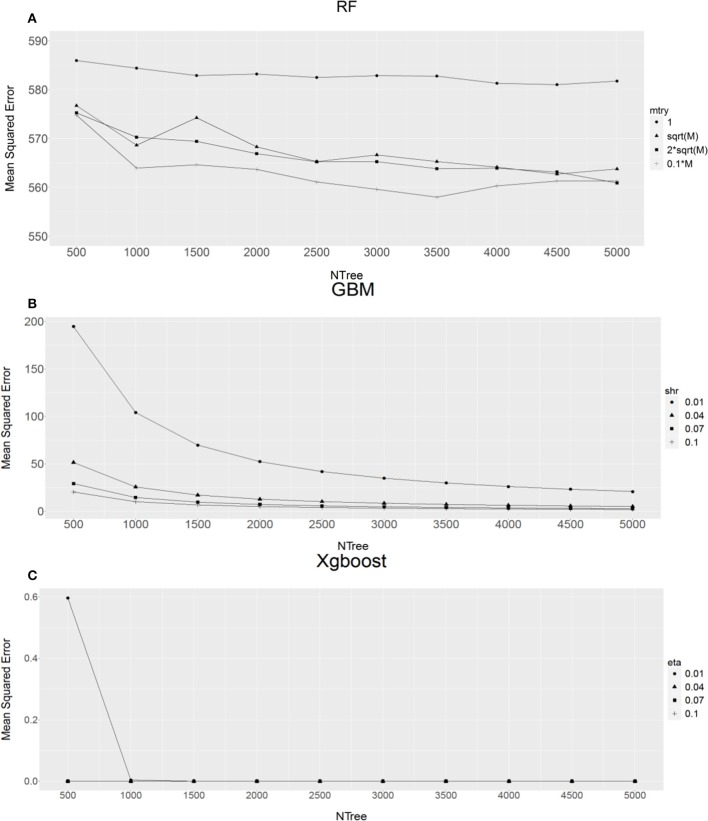
The relationship between different combinations of parameters and mean squared error for **(A)** Random Forest (RF), **(B)** Gradient Boost Machine (GBM) and **(C)** Extreme Gradient Boosting machine (XgBoost). M, total number of SNPs; mtry, number of markers chosen to form a tree, shr for GBM, eta for XgBoost). X-axis refers to the forest tree size (NTree).

The results from an initial examination of the combination of various parameters in individual methods using the bull population are shown in Figure 1.

For RF analyses, when comparing the average MSE values from four different sized markers (mtry), as expected, single marker (mtry = 1) analysis (Figure 1A) produced the highest MSE values, this then followed by sqrt(*M*) (*M* is total number of SNPs, sqrt(*M*) being the default value suggested by RF method) or 2^*^sqrt(*M*). Using 10% of total markers (0.1 × *M*, Figure 1A) had the lowest MSE values. Therefore, 10% of total markers was an obvious choice. In addition, it seems that RF analysis reached a stable status with the forest tree size Ntree ≥ 2,500. This suggests that the RF analysis with Ntree ≥ 2,500 and mtry = 0.1 × *M* should produce precise estimates of SNP VIM values.

For GBM (Figure 1B) and XgBoost (Figure 1C), it can be seen that when Ntree ≥ 2,000, regardless of learning rate value *shr* (GBM) or *eta* (XgBoost), the MSE value became very stable. Therefore, we chose Ntree = 2,000 and *shr* = 0.1 and *eta* = 0.1 for subsequent GBM and XgBoost analyses respectively. The reason for choosing the learning rate of 0.1 for *shr* and *eta*, instead of a much smaller value, is that the smaller the value the longer the program takes to run. In addition, Friedman (2002) suggested that a learning rate of ≤0.1 would lead to better generalization.

### Genome-wide identification of important SNPs

**Figure 2:**
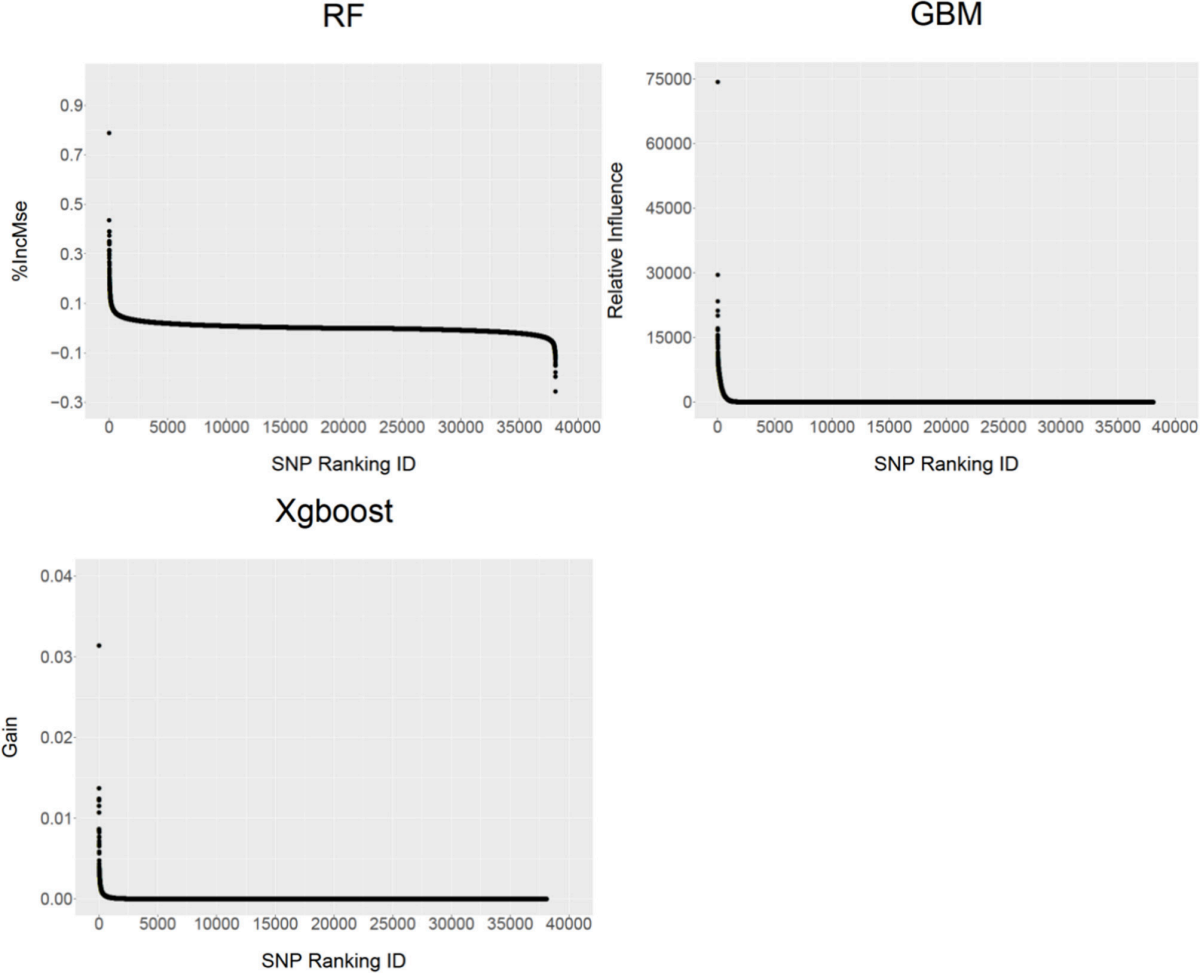
The distribution profiles of ranked SNP variable importance (VIM) values from RF (%IncMSE), GBM (Relative Influence) and XgBoost (Gain).

Unlike parametric models (e.g., a linear mixed model) for GWAS in which the analysis generally provides the parameter estimates such as individual SNP allele substitution effect and a corresponding significance *P* value, the non-parametric models provide SNP VIM values to indicate the contributions of individual SNPs to the MSE. Figure 2 shows the distribution profiles of the VIM values of the ranked SNPs (from the most important to the least important ones) for RF, GBM and XgBoost analyses respectively. The larger the SNP VIM value, the more important a SNP is. As expected, the majority of the SNPs were found to either have very small positive influence or no effect on the VIM values (%IncMse) in RF. In both GBM and XgBoost, there were the SNPs either with very small positive effects or no effect at all. Across three methods, there were 18,453 (48.5%), 16,600 (43.6%), and 9,122 (24%) SNPs identified with positive importance values on the predicted MSE for RF, GBM and XgBoost respectively (Figure 2). In RF, a total of 16,660 SNPs (43.7%) were also found to have negative %InMSE values, corresponding to the lower end of the distribution (Figure 2).

**Figure 3:**
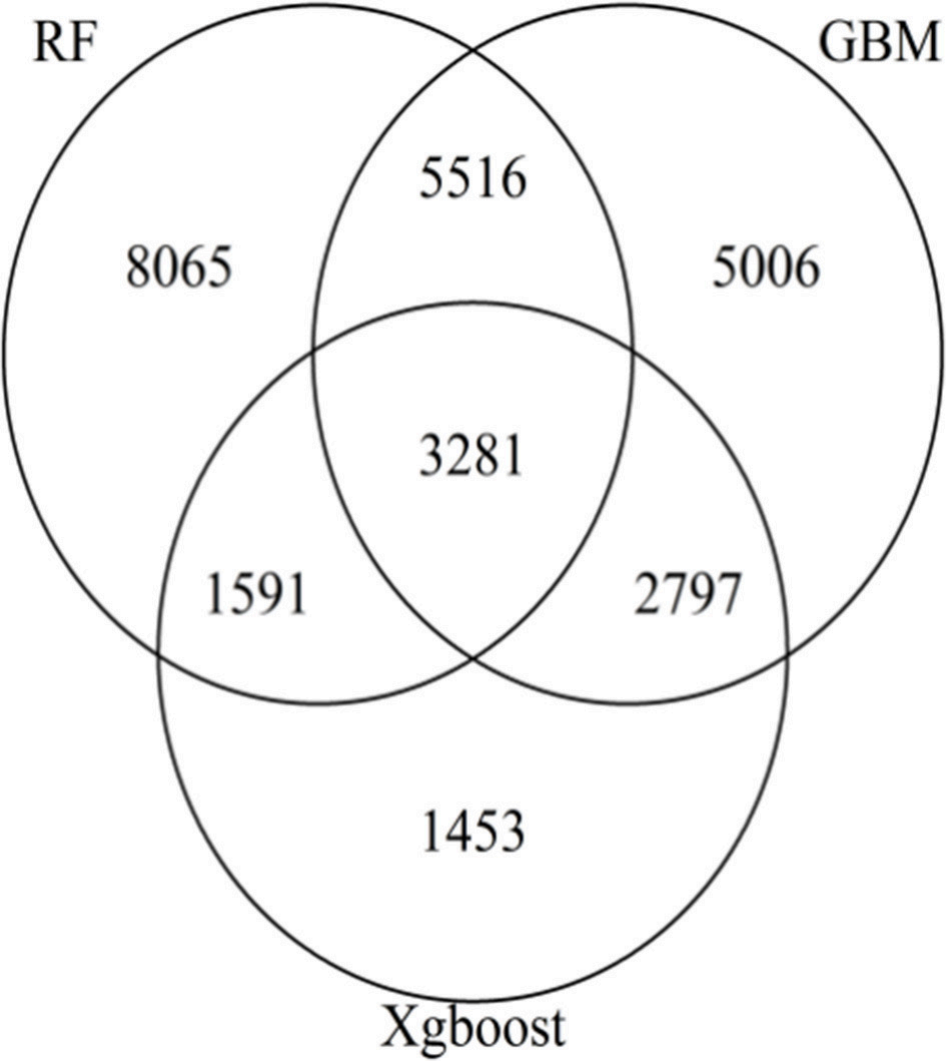
Venn Diagram showing the number of SNPs with non-zero variable importance values for RF, GBM, and XgBoost. Each area of the circle represents the number of SNPs identified by the methods. The areas of intersection of circles represent the number of overlapping SNPs of two or three methods.

The Venn diagram (Figure 3) generated with the SNPs with positive VIM values in either one of the three methods revealed a total of 3,281 SNPs as common markers across three methods. The pair-wise comparison reveals that there were 5,516, 2,797 and 1,591 common SNPs between RF and GBM, between GBM and XgBoost, and between RF and XgBoost, respectively.

**Figure 4:**
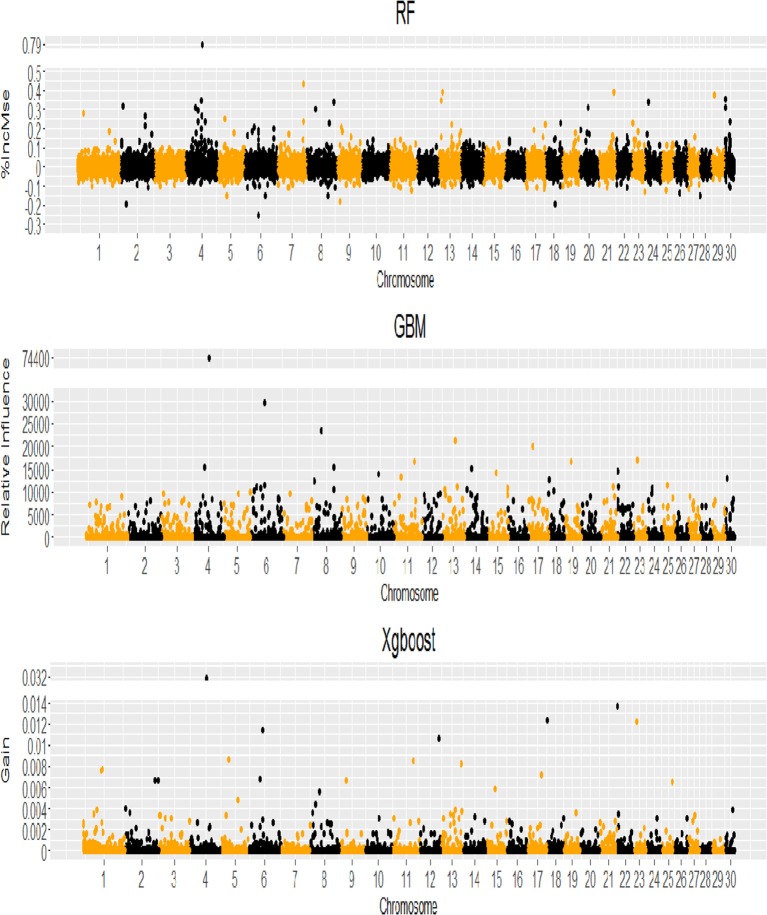
Genome-wide profile of SNP variable importance values for RF, GBM and XgBoost analyses (VIM = %IncMse for RF, Relative Influence for GBM, Gain for XgBoost).

When the genome locations of the SNPs with positive VIM values (see Figure 4) were examined, we found that although the three machine learning methods had different SNP VIM profiles and the top ranking SNPs were scattered across the whole genomes rather than at particular chromosomes, all three methods identified the same SNP with the highest VIM value. It was ARS-BFGL-NGS-1712 mapped to gene *BMPER* (BMP binding Endothelial Regulator) on BTA4. A literature search found that *BMPER* played vital roles in adipocyte differentiation, fat development and energy balance in humans and mice (Zhao et al., 2015). The SNP was a very good candidate for selecting increased body weight and rump length in cattle (Zhao et al., 2015).

When comparing the top 20 SNPs from each of the three methods (Table 1), it was found that other than the SNP mentioned above (ARS-BFGL-NGS-1712 on BTA4), the SNP Hapmap25906-BTA-159707 on BTA8 was also identified in all three methods. There was one SNP Hapmap39284-BTA-70361 (SNP_ID 7315) on BTA4 identified with both RF and GBM, and five SNPs (Hapmap35781-SCAFFOLD166430_3864 on chromosome 6, ARS-BFGL-NGS-85521 on Chromosome 11, Hapmap43561-BTA-36544 on Chromosome 15, ARS-BFGL-NGS-84222 on Chromosome 22 and ARS-BFGL-NGS-86109 on Chromosome 23) in GBM and XgBoost. The results indicate that the similarity was higher between GBM and XgBoost than between RF and GBM.

**Table 1:** The list of top 20 ranking SNPs from Random Forests (RF), Gradient Boosting Machine (GBM) and Extreme Boosting Method (XgBoost).

**Method**	**Rank**	**Chr**	**SNPid**	**Marker name**	**Position (bp)**	**%IncMse**	**Distance to gene**	**Gene name**
RF	1	4	7574	ARS-BFGL-NGS-1712	63,120,929	0.79	0	BMPER
	2	7	13739	ARS-BFGL-NGS-59783	101,669,109	0.44	558,040	TMEM157
	3	21	32659	BTA-119902-no-rs	63,088,634	0.3943	56,593	PAPOLA
	4	13	22207	ARS-BFGL-NGS-10111	15,828,258	0.39	156,328	GATA3
	5	29	38788	Hapmap60043-rs29009948	11,619,866	0.37	0	DLG2
	6	30	39467	BTA-24571-no-rs	4,845,661	0.35	1,117	LAMP2
	7	13	22149	Hapmap54284-ss46526494	10,747,684	0.35	0	SNRPB2
	8	4	7512	ARS-BFGL-NGS-42679	60,157,977	0.35	126,961	GPR141
	9	24	34777	BTA-18966-no-rs	10,631,945	0.34	116,602	CDH19
	10	8	15621	ARS-BFGL-NGS-2393	105,195,851	0.34	0	COL27A1
	11	2	2752	BTA-48707-no-rs	12,087,975	0.32	147,733	LOC787276
	12	30	39466	Hapmap49542-BTA-24574	4,821,238	0.31	25,540	LAMP2
	13	20	31115	Hapmap57531-rs29013890	34,817,221	0.31	201,791	Drosophila
	14	4	7152	ARS-BFGL-NGS-119322	38,573,157	0.31	0	CACNA2D1
	15	8	14488	Hapmap25906-BTA-159707	37,471,009	0.30	178,230	MGC127919
	16	4	7315	Hapmap39284-BTA-70361	48,408,626	0.29	26,562	PRKAR2B
	17	1	309	BTB-01668820	22,590,942	0.28	38,032	LOC788801
	18	2	4080	BTA-48498-no-rs	1.03E+08	0.27	18,075	LOC782360
	19	4	7537	BTB-00192005	61,542,387	0.26	65,408	EEPD1
	20	5	8940	ARS-BFGL-NGS-12166	32,310,418	0.25	0	ASB8
**Method**	**Rank**	**Chr**	**SNPid**	**Marker name**	**Position (bp)**	**Relative influence**	**Distance to gene**	**Gene name**
GBM	1	4	7574	ARS-BFGL-NGS-1712	63,120,929	74352.15	0	BMPER
	2	6	11006	BTB-01845289	53,099,102	29492.17	1,089,822	PCDH7
	3	8	14488	Hapmap25906-BTA-159707	37,471,009	23355.83	178,230	MGC127919
	4	13	22769	BTB-01497093	51,254,301	21264.61	0	LOC529535
	5	17	27548	BTB-00674231	14,814,012	20067.43	60,421	GAB1
	6	23	34003	ARS-BFGL-NGS-86109	16,086,032	17149.69	97,957	TRERF1
	7	11	20254	ARS-BFGL-NGS-85521	78,708,174	16709.08	0	SDC1
	8	19	29905	ARS-BFGL-NGS-31311	26,881,051	16680.46	10,528	RABEP1
	9	4	7315	Hapmap39284-BTA-70361	48,408,626	15590.51	26,562	PRKAR2B
	10	8	15289	ARS-BFGL-NGS-116926	85,880,449	15358.83	0	SUSD3
	11	14	23795	ARS-BFGL-NGS-43648	22,610,144	15084.98	59,217	PCMTD1
	12	22	32802	ARS-BFGL-NGS-84222	531,301	14695.74	0	ECOP
	13	15	25294	Hapmap43561-BTA-36544	36,755,580	14383.39	0	SOX6
	14	10	18018	Hapmap32096-BTA-150413	46,199,529	13796.55	42,360	HERC1
	15	11	19437	BTB-00466621	23,200,575	13183.37	310,170	SLC8A1
	17	18	28553	BTB-01040984	3,080,400	12577.42	97,205	TERF2IP
	18	8	14076	BTA-44195-no-rs	9,448,959	12473.5	0	KIF13B
	19	25	35880	ARS-BFGL-NGS-102269	17,222,665	11622.23	0	GDE1
	20	6	11003	Hapmap35781-SCAFFOLD166430_3864	53,022,829	11552.02	1,013,549	PCDH7
	30	16	39548	ARS-BFGL-NGS-114986	18,377,697	13034.04	28,006	FAM122B
**Method**	**Rank**	**Chr**	**SNPid**	**Marker name**	**Position (bp)**	**Gain**	**Distance to gene**	**Gene name**
Xgboost	1	4	7574	ARS-BFGL-NGS-1712	63,120,929	0.0314	0	BMPER
	2	22	32802	ARS-BFGL-NGS-84222	531,301	0.0137	0	ECOP
	3	18	28525	ARS-BFGL-NGS-21711	1,137,609	0.0124	111,580	UQCRFS1
	4	23	34003	ARS-BFGL-NGS-86109	16,086,032	0.0122	97,957	TRERF1
	5	6	11003	Hapmap35781-SCAFFOLD166430_3864	53,022,829	0.0115	1,013,549	PCDH7
	6	12	21836	BTA-31284-no-rs	83,024,081	0.0107	7,854	KDELC1
	7	5	8919	Hapmap47089-BTA-73292	30,114,907	0.0087	11,765	AQP2
	8	11	20254	ARS-BFGL-NGS-85521	78,708,174	0.0085	0	SDC1
	9	13	23245	ARS-BFGL-NGS-115682	78,901,415	0.0083	0	TMEM189
	10	1	1165	Hapmap38109-BTA-36588	74,581,903	0.0077	0	ATP13A4
	11	1	1088	ARS-BFGL-NGS-118306	69,244,252	0.0076	0	LOC540675
	12	17	28137	Hapmap56365-rs29022398	55,884,612	0.0072	10,824	ORAI1
	13	6	10851	Hapmap43677-BTA-76003	43,772,388	0.0068	0	LOC539625
	14	2	4626	ARS-BFGL-NGS-102755	135,347,580	0.0067	71,006	ACTL8
	15	2	4390	ARS-BFGL-NGS-84506	121,476,153	0.0066	10,154	AK2
	16	9	16165	ARS-BFGL-NGS-58796	28,400,169	0.0066	143,926	LOC785633
	17	25	36203	ARS-BFGL-NGS-10694	36,067,715	0.0066	2,310	PLOD3
	18	15	25294	Hapmap43561-BTA-36544	36,755,580	0.0059	0	SOX6
	19	8	14488	Hapmap25906-BTA-159707	37,471,009	0.0057	178,230	MGC127919
	20	5	9476	BTB-01456593	76,691,828	0.0048	770	SYT10

*Chr, chromosome Number; SNPid, SNP identification number; Marker name, SNP name; Rank, SNP ranking; Distance to gene, Distance to the nearest gene*.

### Gene ontology (GO) enrichment analysis

Tables 2, 3 present the results from the GO Enrichment analyses of top 3,000 SNPs or all SNPs with positive VIM values from each method, using the *Bos taurus* Reference from the PANTHER program. When the biological functions of the genes closest to the top 3,000 SNPs (Table 2) or the SNPs with the positive VIM values (Table 3) were examined, we found that these genes were primarily involved in the development, system development, visual perception, nervous system development and cellular activity (Table 2, *P* < 0.0001). The evidence was much stronger for the genes near all the SNPs with positive VIM values, involving the growth pathways of development process (Table 3, RF: *P* = 1.54^*^10^−7^; GBM: *P* = 2.09^*^10^−8^) and system development (RF: *P* = 5.38^*^10^−7^*;* GBM: *P* = 2.05^*^10^−7^).

**Table 2:** Gene enrichment analysis for top 3,000 SNPs with positive variable importance values from RF, GBM, and XgBoost methods (*P*-value < 0.001 and Fold Enrichment >1).

**Method**	**GO-Slim biological process**	**Reference**	**Uploaded**	**Expected**	**Fold enrichment**	***P*-value**
RF	System development	1013	142	82.71	1.72	1.54E-07
	Developmental process	1835	223	149.82	1.49	5.38E-07
	Heart development	149	37	12.16	3.04	1.56E-06
	Visual perception	185	42	15.1	2.78	1.93E-06
	Nervous system development	619	94	50.54	1.86	3.96E-06
	Sensory perception of sound	70	23	5.72	4.02	9.48E-06
	Muscle organ development	228	46	18.61	2.47	1.22E-05
	Mesoderm development	439	68	35.84	1.9	1.95E-04
GBM	Developmental process	1835	223	144.37	1.54	2.09E-08
	Nervous system development	619	98	48.7	2.01	3.75E-08
	System development	1013	137	79.7	1.72	2.81E-07
	Visual perception	185	39	14.56	2.68	1.66E-05
	Cellular process	8220	735	646.72	1.14	8.84E-04
XgBoost	Nervous system development	619	101	46.65	2.17	3.28E-10
	Developmental process	1835	218	138.28	1.58	5.28E-09
	System development	1013	137	76.34	1.79	1.73E-08
	Cellular process	8220	718	619.43	1.16	3.79E-05
	Visual perception	185	37	13.94	2.65	4.43E-05

*Reference – the number of genes in the reference list, Uploaded – the number of genes in an uploaded list, Expected – number of genes expected in the uploaded list, Fold Enrichment – Ratio of Uploaded/Expected, P value – determined by the binomial statistic test*.

**Table 3:** Gene enrichment analysis for the SNPs with positive variable importance values from RF, GBM, and XgBoost methods (*P*-value < 0.001 and Fold Enrichment > 1).

**Method**	**Gene annotation category**	**Reference**	**Uploaded**	**Expected**	**Fold enrichment**	***P*-value**
RF	Developmental process	1835	675	511.48	1.32	4.91E-11
	Cellular process	8220	2548	2291.21	1.11	4.13E-10
	Nervous system development	619	246	172.54	1.43	1.21E-05
	Visual perception	185	93	51.57	1.8	2.78E-05
	Anatomical structure morphogenesis	160	83	44.6	1.86	3.76E-05
	Mesoderm development	439	182	122.37	1.49	4.99E-05
	Intracellular signal transduction	995	362	277.34	1.31	7.96E-05
	System development	1013	367	282.36	1.3	9.74E-05
GBM	Developmental process	1835	597	464.4	1.29	7.01E-08
	Visual perception	185	90	46.82	1.92	2.77E-06
	Cellular process	8220	2275	2080.33	1.09	3.70E-06
	Nervous system development	619	229	156.66	1.46	5.17E-06
	Cell-cell signaling	449	175	113.63	1.54	9.30E-06
	System development	1013	344	256.37	1.34	1.17E-05
	Mesoderm development	439	167	111.1	1.5	8.14E-05
XgBoost	Developmental process	1835	440	316.8	1.39	7.14E-10
	Nervous system development	619	182	106.87	1.7	2.53E-09
	Visual perception	185	71	31.94	2.22	3.49E-07
	System development	1013	253	174.89	1.45	1.65E-06
	Cellular process	8220	1579	1419.14	1.11	4.61E-06
	Mesoderm development	439	120	75.79	1.58	3.06E-04
	Cell-cell signaling	449	121	77.52	1.56	5.37E-04

*Reference – the number of genes in the reference list, Uploaded – the number of genes in an uploaded list, Expected – number of genes expected in the uploaded list, Fold Enrichment – Ratio of Uploaded/Expected, P-value – determined by the binomial statistic test*.

When converting the genome positions of all of the positive SNPs identified by RF to the human coordinates and checking these against known human biological processes using the GREAT program, we found that there were 16 association terms in our SNP dataset, including AMP catabolic process (*P*-value = 1.22^*^10^−4^), canonical Wnt receptor signaling pathway involved in positive regulation of endothelial cell migration (*P*-value = 1.75^*^10^−4^), positive regulation of cell-cell adhesion (*P*-value = 1.75^*^10^−4^), low density lipoprotein particle mediated signaling (*P*-value = 4.64^*^10^−4)^ and cellular response to lipoprotein particle stimulus (*P*-value = 0.0011).

### Distribution of diagonal elements of genomic relationship matrix (GRM) for different subset of SNPs from the cow population

**Figure 5:**
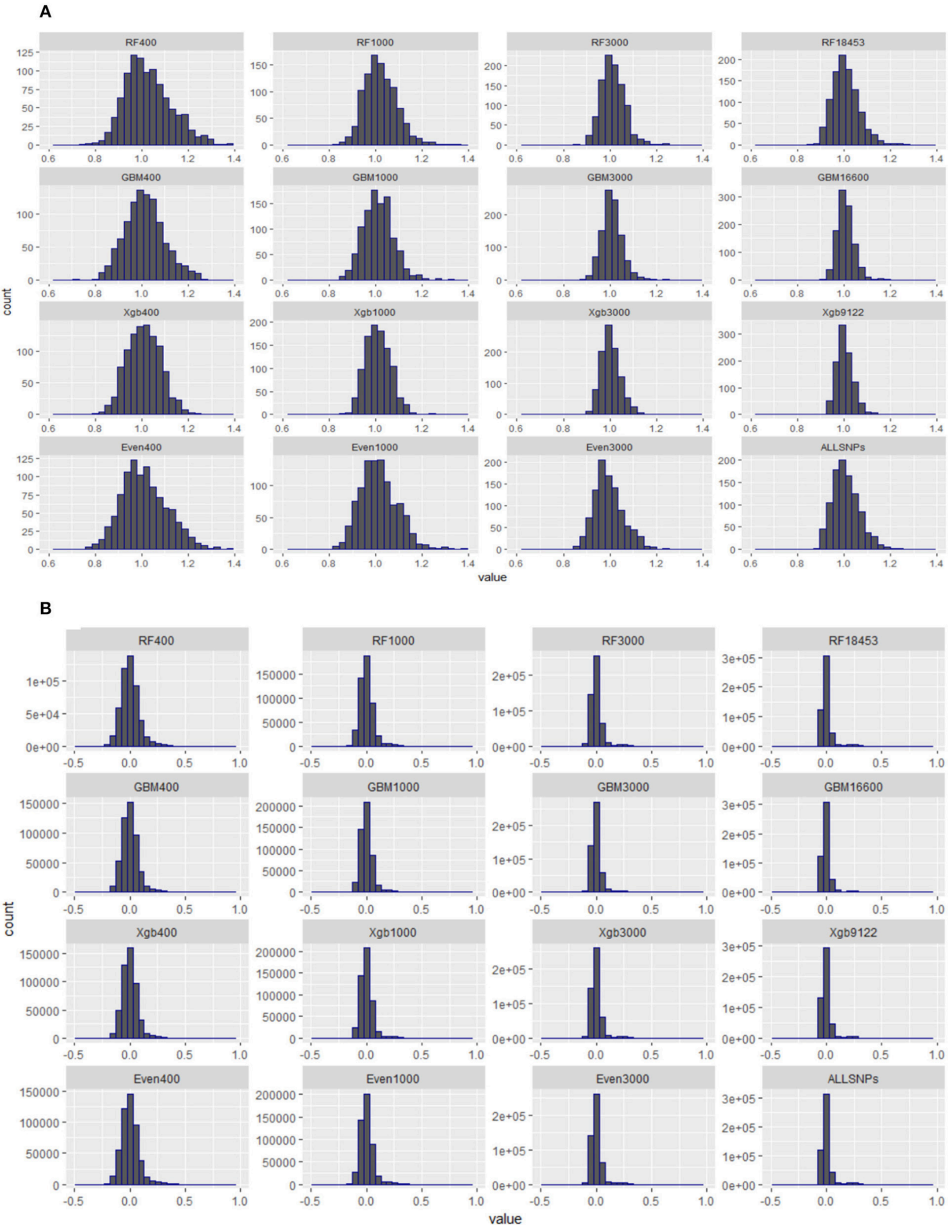
Distributions of diagonal and off-diagonal elements of genomic relationship matrices (GRMs) constructed with different subsets of SNPs from RF, GBM, XgBoost, evenly spaced, or all SNPs of Brahman cow population. **(A)** Diagonal elements of genomic relationship matrices. **(B)** Off-Diagonal elements of genomic relationship matrices.

**Figure 6:**
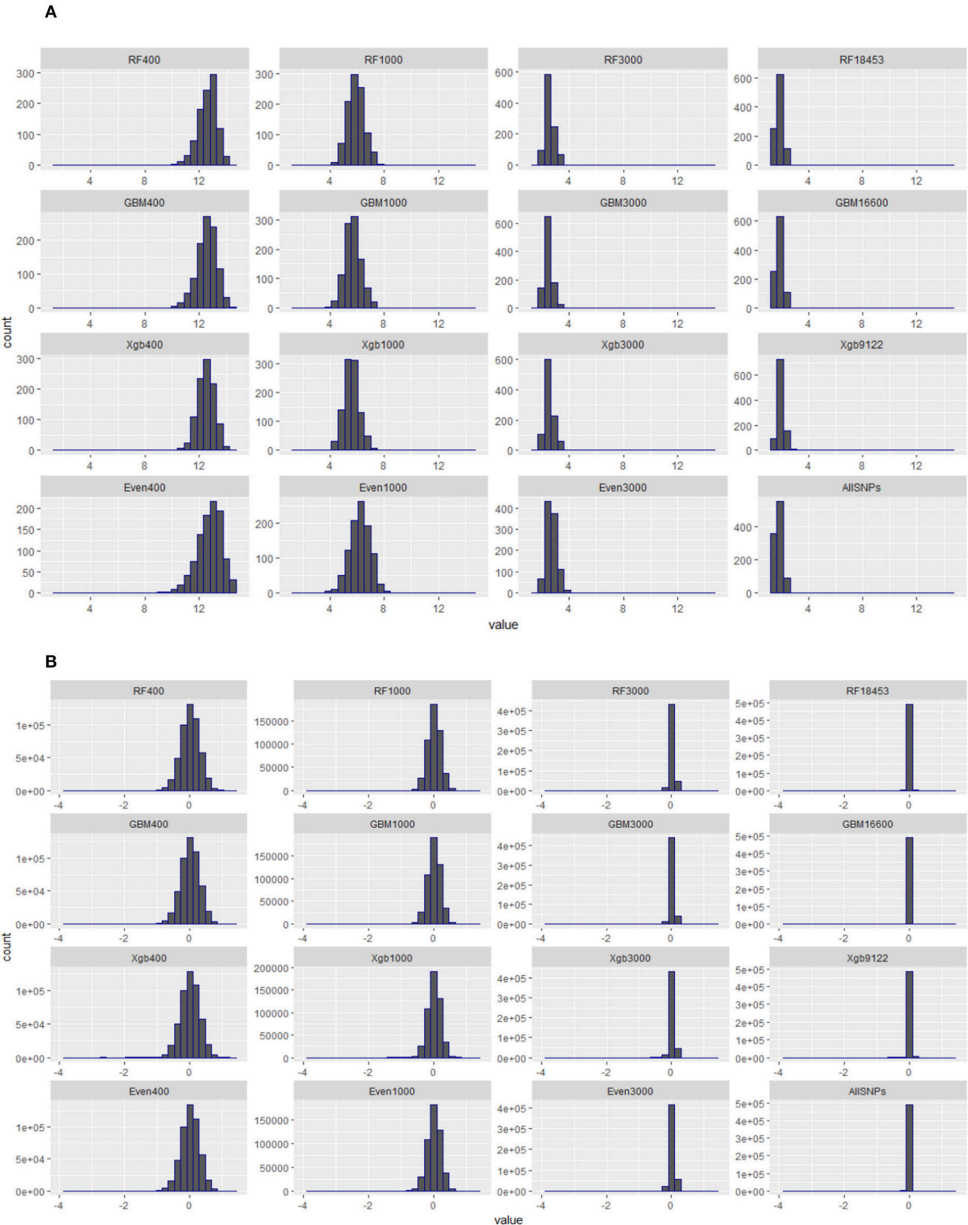
Distributions of diagonal and off-diagonal elements of inversed genomic relationship matrices (inversed-GRMs) constructed with different subsets of SNPs from RF, GBM, XgBoost, evenly spaced, or all SNPs of Brahman cow population. **(A)** Diagonal elements of inversed genomic relationship matrices. **(B)** Off**-**diagonal elements of inversed genomic relationship matrices.

Figure 5 presents the distributions of the diagonal (a) and off-diagonal (b) elements of GRMs constructed using the subsets of SNPs from the Brahman cow population. From Figure 5A it can be seen that the diagonal elements of all GRMs followed a normal distribution, regardless of the sources of the subsets came from, all centered at 1. In fact all GRMs had no distinct multiple peaks suggesting no evidence of hidden sub-population structures in the cow population. In general the off-diagonal elements of all GRMs (Figure 5B) were centered at 0, with a much wider distribution range for the subsets of SNPs either 400 or 1,000. When investigating the diagonal elements of inversed GRMs (Figure 6A), we found that the distributions of diagonal elements from the subsets of SNPs with < 3,000 had significantly larger ranges than those of all SNPs (see the graph named “ALLSNPs” in Figure 6). For example, the average of diagonal elements of the inversed GRM from RF400 (Figure 6) was 12.62 (with a standard deviation STD = 0.698), with a range from 9.89 to 14.55. The average of inversed GRM from RF1000 was 5.90 (STD = 0.61) with a range from 4.05 to 7.70, while the corresponding value from all SNPs was 1.79 (STD = 0.25) with a range of 1.12–2.73. The deflation was even larger for the evenly spaced markers, e.g., Even400 and Even1000.

### Validation of a subset of SNPs using the cow population–estimates of genetic variance

Table 4 shows the REML estimates of additive genetic variances ($\sigma_{\text{a}}^{2}$), residual variances ($\sigma_{\text{e}}^{2}$), total phenotypic variances ($\sigma_{\text{p}}^{2}$) and heritability (h^2^) of live weight in the cow population for a subset of 400, 1,000, 3,000 SNPs and the SNPs with positive VIM values identified by RF, GBM or XgBoost respectively. The same estimates are also given for the evenly spaced 400, 1,000, 3,000 and all the 38,082 SNPs in Table 4.

**Table 4:** Heritability (h^2^) and additive genetic variances ($\sigma_{a}^{2}$) explained by different subsets of SNPs from Brahman cow population.

**Method**	**$\sigma_{a}^{2}$**	**$\sigma_{e}^{2}$**	**$\sigma_{p}^{2}$**	**h^2^**	**% $\sigma_{a}^{2}$ of all SNPs**
RF400	36.37 (16.96)	563.14 (28.96)	599.51 (27.47)	0.061 (0.028)	48.47
RF1,000	45.99 (20.94)	553.36 (30.64)	599.35 (27.47)	0.077 (0.035)	61.29
RF3,000	67.01 (27.79)	533.00 (33.67)	600.02 (27.63)	0.11 (0.046)	89.30
RF18,453†	71.20 (31.70)	529.11 (36.42)	600.32 (27.64)	0.12 (0.052)	94.88
GBM400	39.99 (17.56)	559.71 (28.98)	599.69 (27.52)	0.067 (0.029)	53.29
GBM1,000	61.87 (23.71)	537.93 (31.02)	599.80 (27.66)	0.10 (0.039)	82.45
GBM3,000	82.07 (30.70)	518.64 (34.60)	600.71 (27.83)	0.14 (0.050)	109.37
GBM16,600†	73.57 (32.25)	526.86 (36.62)	600.37 (27.67)	0.12 (0.053)	98.04
XgBoost400	22.28 (15.75)	578.30 (29.64)	600.57 (27.37)	0.037 (0.026)	30.36
XgBoost1,000	35.01 (20.73)	565.45 (31.41)	600.46 (27.43)	0.058 (0.035)	46.66
XgBoost3,000	40.56 (23.45)	559.66 (32.79)	600.22 (27.42)	0.068 (0.039)	54.05
XgBoost9,122†	65.75 (29.30)	534.18 (34.94)	599.63 (27.59)	0.11 (0.049)	87.62
Even400	20.46 (15.25)	580.42 (29.52)	600.88 (27.39)	0.034 (0.026)	27.27
Even1,000	32.42 (19.82)	568.07 (31.13)	600.48 (27.40)	0.054 (0.033)	43.20
Even3,000	53.85 (25.77)	546.55 (33.18)	600.40 (27.56)	0.090 (0.043)	71.76
All SNPs (38,082)	75.04 (32.43)	525.02 (36.77)	600.06 (27.64)	0.125 (0.054)	100.00

$\sigma_{e}^{2}$ – residual variance; $\sigma_{p}^{2}$ – total phenotypic variance.

Standard errors are given in parentheses.

*RF – Random Forests; GBM – Gradient Boosting Machine, XgBoost – Extreme Gradient Boosting; Even – evenly spaced along the genome; The SNPs with positive variable importance values*.

It is clear that in comparison to the estimates from all SNPs (last row in Table 4), the h^2^ estimates (0.11–0.14 with standard error of 0.046–0.053) from the top 3,000 and the SNPs with positive VIM values from RF (18,453 SNPs) and GBM (16,600 SNPs) were very close to the value of using all SNPs (0.125 ± 0.054, Table 4). Across all three machine learning methods, the genetic variances explained by the top 3,000 SNPs or the SNPs with positive VIM values from RF and GBM were more than 89% of the total genetic variance explained by all 38,082 SNPs (see the last column of Table 4). Surprisingly, the top 400 or 1,000 SNPs from RF and GBM also contributed to a substantial amount of genetic variance in the trait, e.g., 48.47% (RF400), 53.29% (GBM400), 61.29% (RF1000), and 82.45% (GNM1000). Of the three methods, the GBM performed particularly well in the cases of 1,000 or 3,000 or the SNPs with positive VIM values, where the genetic variance estimates ($\sigma_{\text{a}}^{2}$) were > 82% that of using all 38,082 SNPs (Table 4).

When examining the results from 400, 1,000, or 3,000 SNPs that were randomly chosen but evenly spaced across the genome (Table 4, with the prefix “Even”), the heritability and genetic variances explained by these SNPs were significantly less than (< 71.76%) of those from all SNPs. When comparing these results of evenly spaced SNPs with those top ranking SNPs (400, 1,000, or 3,000) from RF, GBM and XgBoost, the estimates of genetic variance explained by the evenly spaced SNPs were markedly smaller than those from RF and GBM (Table 4). However, the performance of the subsets of SNPs from Xgboost was similar to those of evenly spaced marker sets.

### Accuracy of prediction of GEBVs

Table 5 shows the average estimated prediction accuracy of GEBVs when using a subset of SNPs in an additive genomic model and a random split five-fold cross-validation scheme in the cow population. In comparison to the results from an additive genomic model using all 38,082 SNPs (last row in Table 5, named All SNPs), the prediction accuracies of the subsets of SNP markers (3,000 or all positive VIM SNPs) chosen by RF or GBM had similar values to that of the whole SNP panel. Of all three methods, GBM had the most superior performance and was then followed by RF and XgBoost. The average prediction accuracy values across 400, 1,000 and 3,000 SNPs were 0.38 (±0.0268) for RF, 0.42 (±0.040) for GBM, and 0.26 (±0.051) for XgBoost. Remarkably, the prediction accuracies from 1,000 (0.42 ± 0.14) and 3,000 (0.46 ± 0.072) SNPs from GBM were the same or slightly better than that of 16,600 SNPs (0.42 ± 0.11), although not significantly.

**Table 5:** Average accuracy of genomic prediction by different subsets of SNPs from Brahman cow population using a 5-fold cross-validation approach.

**Marker**	**Accuracy**
RF400	0.35 (0.072)
RF1000	0.36 (0.10)
RF3000	0.41 (0.15)
RF18453^†^	0.42 (0.14)
GBM400	0.36 (0.19)
GBM1000	0.42 (0.14)
GBM3000	0.46 (0.072)
GBM16600^†^	0.42 (0.11)
Xgb400	0.20 (0.081)
Xgb1000	0.26 (0.092)
Xgb3000	0.33 (0.13)
Xgb9122	0.39 (0.14)
Even400	0.18 (0.055)
Even1000	0.22 (0.13)
Even3000^†^	0.29 (0.19)
All SNPs	0.43 (0.13)

Standard errors are given in parentheses.

RF – Random Forests; GBM – Gradient Boosting Machine, XgBoost – Extreme Gradient Boosting; Even – evenly spaced along the genome;

† *The SNPs with positive variable importance values*.

Using all SNPs with positive VIM values achieved similar prediction accuracy (e.g., 0.42–RF, 0.42–GBM, 0.39–XgBoost, Table 5) when compared with 0.43 from the whole panel. The results suggest that when it comes to the genomic prediction of breeding values, more SNPs in a model do not necessarily translate to a better accuracy. In fact, they may have added more background noises and created more prediction errors than a small number of SNPs that capture the main effects of individual SNPs, SNP-SNP interactions and non-linear relationships.

When comparing the accuracies of the evenly spaced SNP subsets (400, 1,000, and 3,000) with those from three machine learning methods (Table 5), all subsets of SNPs from RF, GBM and Xgboost outperformed those of evenly chosen SNPs, especially RF and GBM. It can be seen that the accuracy values from GBM, 0.36 (GBM400) and 0.42 (GBM1000), were almost double the amount of the evenly spaced SNPs.

### Efficiency of computational time of RF, GBM, and XgBoost

**Figure 7:**
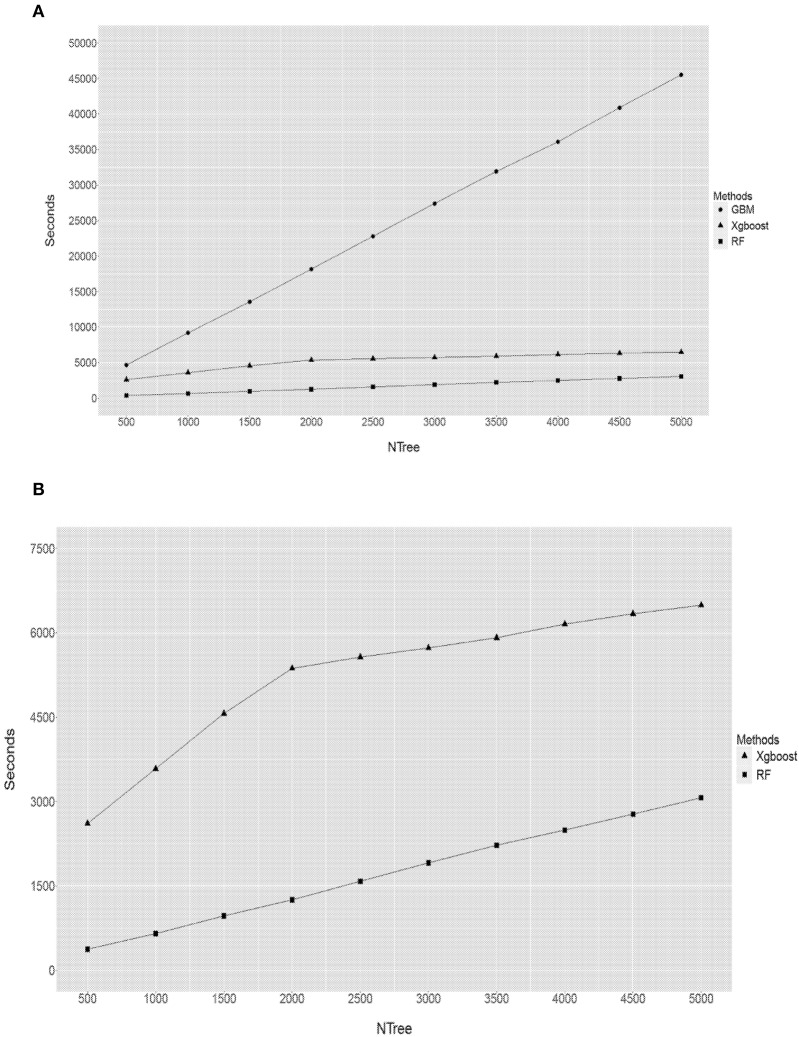
Comparison of computational times undertaken by RF, GBM and XgBoost. Y axis refers to the seconds, X axis refers to the size of forest trees (NTree). **(A)** RF vs. GBM vs. XbBoost **(B)** RF vs. XbBoost.

When comparing the computational time (in terms of seconds) each method had taken to complete an analysis (Figure 7), it is obvious that it depends on the input parameters. For a given discovery population size of 1,097 animals and the total number of SNPs of 38,082, the size of forest trees (Ntree) had the largest impact on the computational time (Figure 7). This is specially the case for the GBM. For example, when the Ntree = 5,000, GBM used about 45,000 s (12.5 h) to finish, while RF took less than an hour and XgBoost less than 2. This is expected as the GBM proceeds through a step-wise of assembling many “weak learners” to build a predictive model and it does not permit parallel computations, while both RF and XgBoost can build decision trees via parallel processes. Therefore the superior performance of GBM was at the cost of an extensive computational time.

## Discussion

Genomic prediction and selection is one of post-genome-era applications that revolutionize genetic improvement programs. Low-density SNP panels can offer a cost effective solution for broad spectra applications of genomic selection programs if subsets of SNPs with biological relevance can be effectively identified to provide high accuracy of genomic prediction of breeding values. While the concept of using low density SNP panels associated with a phenotype as a cost-effective solution for genomic selection has been explored in a number of studies (e.g., Habier et al., 2009; Ogawa et al., 2014), one common recommendation was to select subsets of the markers (e.g., 3,000–6,000) evenly-spaced across the genome for genomic prediction. One of the reasons for selecting equally-spaced markers across traits was to overcome the issue with a subset of SNPs specific to the trait of interest only (Habier et al., 2009; Ogawa et al., 2014). In our study, for a given size of SNP panel (38,082), we found that using the subset of 3,000 SNPs evenly spaced in number across the genome only explained about 71.8% of total additive genetic variance of all the SNPs. This was in vast contrast to the additive genetic variances explained by the 3,000 SNPs selected by the machine learning methods—RF (89.3%) and GBM (109.4%). Moreover, the accuracy of genomic prediction using 3,000 SNPs from RF (0.413) or GBM (0.461) was similar to the value of using whole panel (0.425), while the value being 0.29 for 3,000 evenly spaced SNPs. These results indicate that unless the number of the randomly selected but evenly spaced SNPs is very large, the genomic prediction of low density panel could suffer significant loss of power. This is largely due to the fact that some of the randomly selected but evenly spaced SNPs had small or no effects on the live weight, therefore did not contribute much to the additive genetic variance. In contrast, the top ranking SNPs identified by the machine learning methods had significant influences on the phenotype and hence explained the large proportion of the additive genetic variance. In addition, one of the most important features RF produces is the list of SNPs with negative VIM values indicating the problematic SNPs and highlighting the need of pre-screening to remove these SNPs from the genomic prediction.

Our results from the gene ontology (GO) enrichment analysis clearly indicate that the machine learning methods are efficient methods in identifying a subset of SNPs (e.g., 3,000) with direct links to candidate genes affecting the growth trait. These results could largely contribute to the fact that the machine learning methods captured complex SNP-SNP interactions and non-linear relationships. Therefore, they produced much smaller residual variance, hence, resulted in an increased genetic variance and heritability values.

In supervised learning methods, a prediction error of an algorithm is comprised two parts—a variance and a bias. According to Dietterich and Kong (1995), “the bias of a learning algorithm (for a given learning problem and a fixed size m for training sets) is the persistent or systematic error that the learning algorithm is expected to make when trained on training sets of size *m*.” A goal of a learning algorithm is to minimize both statistical bias and variance. In RF each individual decision tree that is formed with *mtry* SNPs is renowned to be prone to an overfitting prediction error, caused by a high variance and a low bias of an individual tree. However, by using a large number of un-pruned decision trees (i.e., through resampling the data over and over again) to form a forest, the prediction error can be reduced through reducing the variance component (Hastie et al., 2009). While in GBM, a prediction error is due to a low variance and a high bias of a “weak learner.” However, a boosting process improves both bias (through assembling many “weak learners” sequentially and using the weighted sum of predictions of individual trees to reduce the bias) and the variance (by combining many models, Hastie et al., 2009). Therefore in general GBM outperforms RF. In comparison to GBM, XgBoost has more options to choose for regularization to further improve overfitting problems (Chen and He, 2015). Therefore, the performance of XgBoost is expected to be better than GBM. We did observe that the genes close to the top 3,000 SNPs identified by XgBoost had relatively higher P values in the gene enrichment analysis than the ones from GBM and RF. However, when applying the top 3,000 SNPs identified from each method in an additive genomic model for the prediction of GEBVs, surprisingly we see that GBM outperformed XgBoost in the prediction accuracy. This could be due to the fact that there were 18 parameters requiring pre-tuning in XgBoost, we only explored different values for two parameters–Ntree (the number of decision trees) and the learning rate *eta*, not the optimal values for the remaining 16 parameters. These results suggest the complicity of XgBoost parameters.

It is a property of the mixed-models applied in genetic (and genomic) evaluation that prediction error variances are proportional to the diagonal elements of the inverse of the relationship matrix (VanRaden, 2008). In general a low-density panel could inevitably result in higher variance in genomic relationship estimates. This high variation could translate into large diagonals of the GRM inverse which in turn results in inflated accuracy estimates (Hill and Weir, 2011). In our study here, in comparison to the results from using all SNPs, the evidence of much increased variances in both genomic relationship matrices (GRMs) and inversed GRMs (Figures 5, 6) was very strong in the cases where the subsets of 400 or 1,000 SNPs were used for genomic prediction of the cow population, regardless of the methods used for selecting the subsets of SNPs. However, the large variances in GRMs diminished as the density of SNPs reached beyond 3,000. Therefore, this suggests that a minimum of 3,000 SNPs would be required to implement genomic selection tools.

It is worth pointing out that the additive genetic variance and heritability values referred to in this study are not the same as the strict definitions of traditional quantitative genetics theory (de los Campos et al., 2015). They should be “genomic variance” and “genomic heritability.” According to de los Campos et al. (2015), “the genomic heritability and the trait heritability parameters are equal only when all causal variants are typed.” Given that the number of true QTLs are unknown and a limited number of SNPs is used, these estimates are biased from the true additive genetic variance and heritability value of the population. These could also impact on our results.

Since all machine learning methods are non-parametric models, these models do not differentiate between fixed environmental effects and random genetic effects. If fixed environmental effects were directly used as covariates, they would be treated as predictor variables as SNPs, then the subset SNP results would be dependent on these fixed effects. Therefore, we pre-adjusted the phenotype the same way as the other studies (Lubke et al., 2013; Waldmann, 2016). However, a GBLUP model is a mixed model in which fixed effects (age and contemporary group effects) can be properly separated from the random genetic effect, hence we used the original phenotype for the GBLUP model in the validation population. It is possible that the accuracy could be different if we also used the pre-adjusted phenotype for the GBLUP analysis in the validation population.

Both linkage disequilibrium (LD) and MAF (minor allele frequency) can systematically impact the variable importance measures used by both RF and GBM (Strobl et al., 2007; Habier et al., 2009; Walters et al., 2012; Lubke et al., 2013; Ogawa et al., 2014; Zhou and Troyanskaya, 2015). Walters et al. (2012) suggested applying a sliding window algorithm that uses overlapping subsets of SNPs chosen from a whole genome association study to assign the SNPs with high LD to different subsets to reduce bias in VIM. We did not apply the method in our analyses, as the Manhattan plots from 3 methods (Figure 3) showed that the top ranking SNP markers (e.g., 400, 1,000, and 3,000) were relatively sparsely spaced along the whole genome.

The prediction accuracy of genomic breeding values can be affected by a number of factors, for example, number of animals in a training (or reference) population, heritability of a trait of interest, relationship between training and validation animals (i.e., genetic architecture), length of chromosomes (in Morgans) and the effective population size (Goddard, 2009; Howard et al., 2014). There are a few limitations in this study. Firstly, our training and the validation populations (the Brahman bull and cow populations) were not independent and they were related half-sibs. The accuracy of genomic prediction of breeding values could change when different training and validation populations are used. Therefore caution is needed for the interpretation of our results. Secondly, we only examined a phenotype with the moderate heritability—live yearling weight in beef cattle. Further studies are required to further validate the efficiency of machine learning methods in building low density SNP panels for genomic prediction, for a range of phenotypes with different heritability values under various population sizes. Thirdly, we only investigated the predictability of subsets of top ranking SNPs with the effects on a univariate—live weight. The pleiotropy of the subset SNPs could have the impact on the traits correlated to the live weight. Fourthly, we applied a random 5-fold cross validation scheme, rather than the split of the animals from the same sire families into the same group and no connection between training and validation datasets (i.e., a family-based cross-validation scheme). Therefore the results would be expected to be very different for the family-based cross-validation scheme.

There is no doubt that there are other machine learning methods that can be used for high dimension reduction and efficient selection of subsets of SNPs for low-density SNP panels (e.g., Liang and Kelemen, 2008; Long et al., 2011; Walters et al., 2012; Bermingham et al., 2015), and then apply the panels for genomic prediction of breeding values. The machine learning methods such as GBM have the advantage over parametric methods for its ability in dealing with variable interactions, nonlinear relationships, outliers, and missing values. They can also be used to initially identify a small number of informative SNPs associated with phenotypes and then use these SNPs for the imputation to high density genotypes to further improve the accuracy of genomic prediction.

It is worthwhile to mention that this study here intended to serve as a proof of concept. We have also applied RF as a pre-screening tool for identifying low-density SNPs for genomic prediction in another beef cattle population that consisted of 2,109 Brahman cattle with 651,253 SNP genotypes and found the similar results (Li et al., 2018). One of the limitations for this study is that we only evaluated three machine learning methods for selecting subsets of SNPs for genomic prediction of a single trait, rather than for multiple traits. There is literature available about the application of machine learnings for genomic prediction of multiple traits (He et al., 2016; Paré et al., 2017). However, given complex relationships among multiple traits and SNPs, a vigorous evaluation of three methods for selecting subsets of SNPs affecting multiple traits is beyond the scope of the current study. Tackling multiple traits will be the future work.

The outcomes from this study have a number of potential implementations. For example, (1) using the machine learning methods as a pre-screening tool (or a high-dimension reduction tool) to identify biologically relevant variants from large genome sequence variants of a large population, and then apply subsets for detailed investigation of gene functions or pathways or genomic prediction of future generations; (2) Building large reference populations by initially genotyping a large SNP panel on part of a population, and then choosing subsets of SNPs to genotype the rest of a population for future genomic selection.

## Conclusions

In this study, using the live weight from Brahman cattle and 38,083 SNPs, we demonstrated that two machine learning methods—RF and GBM, are efficient in identifying potential candidate genes for the growth trait. Using at least 3,000 SNPs with positive VIM values identified by RF and especially GBM achieved the similar estimates of heritability and genomic prediction accuracy of breeding values as those of using all SNPs. The subsets of SNPs (400, 1,000, and 3,000) selected by the RF and GBM significantly outperformed those SNPs evenly spaced across the genome. The superiority of GBM performance comes at the expense of longer computational time.

## Data availability statement

The raw data supporting the conclusions of this manuscript, containing 40,184 SNP genotypes and body weight phenotypes from 2,093 Brahman cattle, are part of the Australia Beef CRC project (http://www.beefcrc.com/) and are co-owned with Meat and Livestock Australia. The data can be made available subject to the agreement of the owners. Requests to access the raw dataset should be directed to YL (yutao. li@csiro.au).

## Author contributions

BL performed the data analysis using the machine learning methods in the bull population and provided the information to the manuscript. NZ and Y-GW were involved in the initial programing of RF and GBM methods. AG and AR provided valuable input on the project and assist improving the manuscript. YL provided crucial concepts, supervised the project, conducted the genomic prediction in the cow population and drafted the manuscript. All authors read and approved the final manuscript.

### Conflict of interest statement

The authors declare that the research was conducted in the absence of any commercial or financial relationships that could be construed as a potential conflict of interest.

## Supplementary material

The detailed statistical information on individual GRM and inversed GRM are given in Tables 6 and 7 in Supplementary Material. The estimates of the heritability, variance and genomic prediction accuracy from using each subset of SNPs in individual cross-validation subset are presented in Tables 8 and 9 in Supplementary Material.
