# Supplementary material for: Genomic Prediction of Breeding Values Using a Subset of SNPs Identified by Three Machine Learning Methods
Source: Front Genet. 2018 Jul 4;9:237. doi: 10.3389/fgene.2018.00237 (PMC6039760; doi:10.3389/fgene.2018.00237)
Supplement: Supplementary file 1 [file Table_1.pdf]

**Supplementary Material**

**Supplementary Table S1**. Average diagonal and off diagonal values of genomic relationship matrices (GRMs) constructed from subsets of SNPs of Brahman cow population

| | Diagonal | | | | Off-diagonal | | | |
|---|---|---|---|---|---|---|---|---|
| **Marker** | **Mean** | **STD** | **Min** | **Max** | **Mean** | **STD** | **Min** | **Max** |
| AllSNP | 1.01 | 0.059 | 0.87 | 1.29 | -0.0010 | 0.048 | -0.010 | 0.65 |
| RF400 | 1.03 | 0.10 | 0.73 | 1.37 | -0.0010 | 0.082 | -0.34 | 0.74 |
| RF1000 | 1.02 | 0.071 | 0.83 | 1.34 | -0.0010 | 0.065 | -0.23 | 0.74 |
| RF3000 | 1.01 | 0.051 | 0.86 | 1.25 | -0.0010 | 0.053 | -0.15 | 0.68 |
| RF18453† | 1.01 | 0.058 | 0.87 | 1.28 | -0.0010 | 0.049 | -0.096 | 0.65 |
| GBM400 | 1.01 | 0.085 | 0.73 | 1.40 | -0.0010 | 0.075 | -0.29 | 0.70 |
| GBM1000 | 1.01 | 0.064 | 0.86 | 1.32 | -0.0010 | 0.060 | -0.20 | 0.70 |
| GBM3000 | 1.01 | 0.043 | 0.89 | 1.23 | -0.0010 | 0.052 | -0.15 | 0.68 |
| GBM16600† | 1.01 | 0.036 | 0.92 | 1.18 | -0.0010 | 0.049 | -0.10 | 0.65 |
| Xgb400 | 1.01 | 0.075 | 0.80 | 1.37 | -0.0010 | 0.073 | -0.32 | 0.73 |
| Xgb1000 | 1.01 | 0.054 | 0.86 | 1.25 | -0.0010 | 0.061 | -0.23 | 0.67 |
| Xgb3000 | 1.00 | 0.041 | 0.91 | 1.22 | -0.0010 | 0.053 | -0.14 | 0.64 |
| Xgb9122† | 1.00 | 0.036 | 0.92 | 1.17 | -0.0010 | 0.050 | -0.11 | 0.65 |
| Even400 | 1.01 | 0.10 | 0.77 | 1.43 | -0.0010 | 0.078 | -0.30 | 0.77 |
| Even1000 | 1.01 | 0.081 | 0.82 | 1.38 | -0.0010 | 0.061 | -0.23 | 0.67 |
| Even3000 | 1.00 | 0.063 | 0.86 | 1.25 | -0.0010 | 0.052 | -0.17 | 0.62 |

RF – Random Forests; GBM – Gradient Boosting Machine, XgBoost – Extreme Gradient Boosting; Even – evenly spaced along the genome; † The SNPs with positive variable importance values.

**Supplementary Table S2**. Average diagonal and off-diagonal values of inversed genomic relationship matrices (GRMs) constructed from subsets of SNPs of Brahman cow population

| | **Diagonal** | | | | **Off diagonal** | | | |
|---|---|---|---|---|---|---|---|---|
| **Marker** | **Mean** | **STD** | **Min** | **Max** | **Mean** | **STD** | **Min** | **Max** |
| AllSNP | 1.79 | 0.25 | 1.12 | 2.73 | 0.018 | 0.032 | -1.15 | 0.29 |
| RF400 | 12.62 | 0.70 | 9.89 | 14.55 | 0.007 | 0.29 | -3.64 | 1.48 |
| RF1000 | 5.90 | 0.61 | 4.05 | 7.70 | 0.014 | 0.20 | -2.92 | 0.96 |
| RF3000 | 2.55 | 0.33 | 1.74 | 3.75 | 0.018 | 0.066 | -1.55 | 0.42 |
| RF18453 | 1.85 | 0.26 | 1.17 | 2.85 | 0.018 | 0.035 | -1.17 | 0.31 |
| GBM400 | 12.55 | 0.71 | 10.02 | 14.60 | 0.007 | 0.29 | -3.36 | 1.36 |
| GBM1000 | 5.68 | 0.59 | 3.91 | 7.42 | 0.014 | 0.20 | -2.85 | 0.88 |
| GBM3000 | 2.46 | 0.31 | 1.641 | 3.72 | 0.018 | 0.062 | -1.53 | 0.39 |
| GBM16600 | 1.84 | 0.24 | 1.26 | 2.85 | 0.018 | 0.035 | -1.13 | 0.29 |
| Xgb400 | 12.48 | 0.63 | 9.82 | 14.39 | 0.008 | 0.29 | -3.31 | 1.47 |
| Xgb1000 | 5.57 | 0.56 | 4.002 | 7.29 | 0.014 | 0.19 | -2.97 | 0.88 |
| Xgb3000 | 2.55 | 0.32 | 1.762 | 3.97 | 0.018 | 0.065 | -1.66 | 0.40 |
| Xgb9122 | 1.98 | 0.26 | 1.352 | 3.08 | 0.018 | 0.040 | -1.25 | 1.00 |
| Even400 | 12.77 | 0.91 | 9.09 | 14.95 | 0.007 | 0.284 | -3.79 | 1.18 |
| Even1000 | 6.19 | 0.73 | 3.63 | 8.38 | 0.014 | 0.21 | -3.09 | 0.96 |
| Even3000 | 2.69 | 0.37 | 1.67 | 4.02 | 0.017 | 0.071 | -1.65 | 0.47 |

RF – Random Forests; GBM – Gradient Boosting Machine, XgBoost – Extreme Gradient Boosting; Even – evenly spaced along the genome; † The SNPs with positive variable importance values.

**Supplementary Table S3.** Five-fold cross-validation scheme - individual subset estimates of heritability ($h^2$) and additive genetic variances ($\sigma_a^2$) explained by different subsets of SNPs from Brahman cow population.

| Marker | $\sigma_a^2$ | SD | $\sigma_e^2$ | SD | $\sigma_p^2$ | SD | $h^2$ | SD |
|---|---|---|---|---|---|---|---|---|
| RF400_R1 | 38.66 | 21.31 | 574.75 | 34.18 | 613.41 | 31.52 | 0.063 | 0.035 |
| RF400_R2 | 45.05 | 21.59 | 553.42 | 33.07 | 598.48 | 30.82 | 0.075 | 0.036 |
| RF400_R3 | 31.42 | 19.39 | 559.89 | 32.95 | 591.31 | 30.31 | 0.053 | 0.033 |
| RF400_R4 | 30.21 | 17.75 | 555.51 | 32.14 | 585.72 | 30.01 | 0.052 | 0.030 |
| RF400_R5 | 39.94 | 21.06 | 573 | 33.98 | 612.94 | 31.49 | 0.065 | 0.034 |
| average | 37.06 | 20.22 | 563.31 | 33.26 | 600.37 | 30.83 | 0.062 | 0.034 |
| RF1000_R1 | 57.9 | 28.25 | 555.6 | 36.9 | 613.5 | 31.64 | 0.094 | 0.046 |
| RF1000_R2 | 61.95 | 27.8 | 536.79 | 35.7 | 598.74 | 30.91 | 0.10 | 0.046 |
| RF1000_R3 | 35.84 | 23.08 | 555.29 | 34.93 | 591.13 | 30.27 | 0.061 | 0.039 |
| RF1000_R4 | 35.00 | 21.64 | 551.14 | 34.05 | 586.15 | 30.02 | 0.060 | 0.037 |
| RF1000_R5 | 51.05 | 26.42 | 562 | 36.47 | 613.05 | 31.52 | 0.083 | 0.043 |
| average | 48.35 | 25.44 | 552.16 | 35.61 | 600.51 | 30.87 | 0.080 | 0.042 |
| RF3000_R1 | 78.84 | 35.57 | 534.86 | 40.68 | 613.71 | 31.76 | 0.13 | 0.057 |
| RF3000_R2 | 83.13 | 36.13 | 516.94 | 40.22 | 600.08 | 31.12 | 0.14 | 0.059 |
| RF3000_R3 | 41.49 | 28.44 | 550.04 | 38.07 | 591.53 | 30.29 | 0.070 | 0.048 |
| RF3000_R4 | 64.58 | 31.45 | 522 | 38.07 | 586.57 | 30.24 | 0.11 | 0.053 |
| RF3000_R5 | 72.98 | 33.89 | 540.28 | 40.07 | 613.26 | 31.65 | 0.12 | 0.055 |
| average | 68.20 | 33.10 | 532.82 | 39.42 | 601.03 | 31.01 | 0.11 | 0.055 |
| RF18453_R1 | 80.31 | 39.26 | 533.17 | 43.69 | 613.48 | 31.7 | 0.13 | 0.064 |
| RF18453_R2 | 90.46 | 41.95 | 510.4 | 44.46 | 600.87 | 31.18 | 0.15 | 0.069 |
| RF18453_R3 | 46.38 | 32.07 | 545.07 | 40.38 | 591.45 | 30.29 | 0.078 | 0.055 |
| RF18453_R4 | 60.17 | 33.83 | 526.62 | 40.41 | 586.79 | 30.17 | 0.10 | 0.058 |
| RF18453_R5 | 80.9 | 40.22 | 533.22 | 44.43 | 614.12 | 31.73 | 0.13 | 0.065 |
| average | 71.64 | 37.47 | 529.70 | 42.67 | 601.34 | 31.01 | 0.12 | 0.062 |
| GBM400_R1 | 48.41 | 22.64 | 564.63 | 33.98 | 613.03 | 31.62 | 0.079 | 0.037 |
| GBM400_R2 | 45.19 | 22.19 | 554.25 | 33.4 | 599.44 | 30.88 | 0.075 | 0.037 |
| GBM400_R3 | 28.7 | 19.432 | 562.94 | 33.26 | 591.65 | 30.29 | 0.049 | 0.033 |
| GBM400_R4 | 64.76 | 23.17 | 520.29 | 31.63 | 585.05 | 30.47 | 0.11 | 0.039 |
| GBM400_R5 | 26.97 | 18.84 | 586.32 | 34.16 | 613.29 | 31.37 | 0.044 | 0.031 |
| average | 42.81 | 21.25 | 557.69 | 33.29 | 600.49 | 30.93 | 0.072 | 0.035 |
| GBM1000_R1 | 74.28 | 30.39 | 538.92 | 36.93 | 613.2 | 31.8 | 0.12 | 0.049 |
| GBM1000_R2 | 74.68 | 31.05 | 525.81 | 36.73 | 600.49 | 31.18 | 0.12 | 0.051 |
| GBM1000_R3 | 46.14 | 25.6 | 545.24 | 35.56 | 591.38 | 30.36 | 0.078 | 0.043 |
| GBM1000_R4 | 70.51 | 27.92 | 515.23 | 34.74 | 585.74 | 30.35 | 0.12 | 0.047 |
| GBM1000_R5 | 52.46 | 27.2 | 560.88 | 36.89 | 613.34 | 31.53 | 0.086 | 0.044 |
| average | 63.61 | 28.43 | 537.22 | 36.17 | 600.83 | 31.044 | 0.11 | 0.047 |
| GBM3000_R1 | 85.36 | 36.67 | 528 | 41.06 | 613.36 | 31.79 | 0.14 | 0.059 |
| GBM3000_R2 | 109.13 | 40.92 | 493.01 | 41.81 | 602.14 | 31.57 | 0.18 | 0.066 |
| GBM3000_R3 | 76.96 | 35.03 | 514.91 | 39.82 | 591.87 | 30.62 | 0.13 | 0.059 |
| GBM3000_R4 | 65.91 | 32.77 | 521.1 | 38.91 | 587 | 30.28 | 0.11 | 0.056 |
| GBM3000_R5 | 76.08 | 36.17 | 538.04 | 41.47 | 614.12 | 31.73 | 0.12 | 0.058 |
| average | 82.69 | 36.31 | 519.01 | 40.61 | 601.70 | 31.20 | 0.14 | 0.060 |

| | | | | | | | | |
|---|---|---|---|---|---|---|---|---|
| GBM16600_R1 | 81.66 | 39.45 | 531.68 | 43.72 | 613.34 | 31.71 | 0.13 | 0.064 |
| GBM16600_R2 | 94.34 | 42.57 | 506.69 | 44.66 | 601.04 | 31.24 | 0.16 | 0.070 |
| GBM16600_R3 | 56.83 | 34.58 | 534.77 | 41.32 | 591.6 | 30.38 | 0.096 | 0.059 |
| GBM16600_R4 | 57.06 | 33.27 | 529.79 | 40.22 | 586.84 | 30.16 | 0.097 | 0.057 |
| GBM16600_R5 | 80.19 | 40.38 | 534.09 | 44.57 | 614.27 | 31.74 | 0.13 | 0.065 |
| average | 74.02 | 38.05 | 527.40 | 42.90 | 601.42 | 31.04 | 0.13 | 0.063 |
| Xgb400_R1 | 32.17 | 20.52 | 581.1 | 34.45 | 613.27 | 31.42 | 0.053 | 0.034 |
| Xgb400_R2 | 21.51 | 19.73 | 578.29 | 34.36 | 599.81 | 30.64 | 0.036 | 0.033 |
| Xgb400_R3 | 22.81 | 18.2 | 568.75 | 33.21 | 591.56 | 30.24 | 0.039 | 0.031 |
| Xgb400_R4 | 17.74 | 18.05 | 569.93 | 33.39 | 587.67 | 30.03 | 0.030 | 0.031 |
| Xgb400_R5 | 23.43 | 20.44 | 590.69 | 35.18 | 614.12 | 31.4 | 0.038 | 0.034 |
| average | 23.53 | 19.39 | 577.75 | 34.12 | 601.29 | 30.75 | 0.039 | 0.033 |
| Xgb1000_R1 | 37.1 | 24.83 | 576.07 | 36.73 | 613.18 | 31.4 | 0.061 | 0.041 |
| Xgb1000_R2 | 48.91 | 28.12 | 551.27 | 37.06 | 600.19 | 30.85 | 0.082 | 0.047 |
| Xgb1000_R3 | 36.32 | 24.34 | 555.17 | 35.63 | 591.48 | 30.29 | 0.061 | 0.041 |
| Xgb1000_R4 | 31.09 | 23.66 | 556.44 | 35.46 | 587.53 | 30.08 | 0.053 | 0.041 |
| Xgb1000_R5 | 34.14 | 26.08 | 579.97 | 37.7 | 614.11 | 31.44 | 0.056 | 0.043 |
| average | 37.51 | 25.41 | 563.78 | 36.52 | 601.30 | 30.81 | 0.062 | 0.042 |
| Xgb3000_R1 | 52.68 | 30.07 | 560.22 | 38.94 | 612.9 | 31.47 | 0.086 | 0.049 |
| Xgb3000_R2 | 46.68 | 30.56 | 553.22 | 39.15 | 599.91 | 30.77 | 0.078 | 0.051 |
| Xgb3000_R3 | 32.64 | 26.07 | 558.99 | 37.17 | 591.64 | 30.25 | 0.055 | 0.044 |
| Xgb3000_R4 | 33.32 | 26.11 | 554.11 | 36.95 | 587.43 | 30.07 | 0.057 | 0.045 |
| Xgb3000_R5 | 43.98 | 29.73 | 569.83 | 39.43 | 613.81 | 31.46 | 0.072 | 0.049 |
| average | 41.86 | 28.51 | 559.27 | 38.33 | 601.14 | 30.80 | 0.070 | 0.048 |
| Xgb9122_R1 | 79.99 | 37.4 | 533.04 | 42.12 | 613.03 | 31.7 | 0.13 | 0.060 |
| Xgb9122_R2 | 80.81 | 38.66 | 519.68 | 42.46 | 600.49 | 31.09 | 0.14 | 0.064 |
| Xgb9122_R3 | 54.23 | 32.25 | 537.19 | 39.72 | 591.41 | 30.37 | 0.092 | 0.055 |
| Xgb9122_R4 | 51.65 | 30.83 | 535.21 | 38.78 | 586.86 | 30.14 | 0.088 | 0.053 |
| Xgb9122_R5 | 80.69 | 38.28 | 533.21 | 42.76 | 613.89 | 31.75 | 0.13 | 0.062 |
| average | 69.47 | 35.48 | 531.67 | 41.17 | 601.13 | 31.01 | 0.12 | 0.059 |
| Even400_R1 | 33.1 | 20.55 | 597.38 | 35.06 | 630.48 | 32.32 | 0.053 | 0.033 |
| Even400_R2 | 28.61 | 19.07 | 563.03 | 32.99 | 591.63 | 30.31 | 0.0483 | 0.0328 |
| Even400_R3 | 10.54 | 16.38 | 584.9 | 33.56 | 595.43 | 30.37 | 0.018 | 0.028 |
| Even400_R4 | 13.32 | 16.87 | 561.88 | 32.61 | 575.1 | 29.35 | 0.023 | 0.030 |
| Even400_R5 | 28.6 | 20.41 | 583.86 | 34.56 | 612.46 | 31.37 | 0.047 | 0.0338 |
| average | 22.834 | 18.656 | 578.21 | 33.756 | 601.02 | 30.744 | 0.038 | 0.0318 |
| Even1000_R1 | 39.17 | 24.2 | 590.47 | 36.97 | 629.64 | 32.23 | 0.0629 | 0.039 |
| Even1000_R2 | 35.37 | 24.17 | 556.1 | 35.57 | 591.46 | 30.29 | 0.060 | 0.0417 |
| Even1000_R3 | 16.39 | 21.56 | 579 | 35.86 | 595.39 | 30.38 | 0.028 | 0.037 |
| Even1000_R4 | 35.87 | 23.15 | 538.69 | 34.26 | 574.56 | 29.43 | 0.062 | 0.040 |
| Even1000_R5 | 41.84 | 26.46 | 570.35 | 37.24 | 612.19 | 31.39 | 0.068 | 0.043 |
| average | 33.728 | 23.91 | 566.93 | 35.98 | 600.65 | 30.745 | 0.056 | 0.040 |
| Even3000_R1 | 76.61 | 34.59 | 552.92 | 40.62 | 629.53 | 32.55 | 0.12 | 0.054 |
| Even3000_R2 | 43.13 | 27.06 | 548.07 | 37.2 | 591.2 | 30.31 | 0.073 | 0.047 |
| Even3000_R3 | 36.32 | 28.99 | 559.47 | 38.7 | 595.78 | 30.52 | 0.061 | 0.049 |
| Even3000_R4 | 71.74 | 31.86 | 502.82 | 37.08 | 574.56 | 29.74 | 0.13 | 0.055 |
| Even3000_R5 | 62.86 | 33.12 | 549.67 | 40.02 | 612.52 | 31.58 | 0.10 | 0.054 |
| average | 58.13 | 31.124 | 542.59 | 38.72 | 600.72 | 30.94 | 0.097 | 0.052 |

| | | | | | | | | |
|---|---|---|---|---|---|---|---|---|
| AllSNP _R1 | 85.53 | 40.62 | 527.83 | 44.42 | 613.37 | 31.73 | 0.14 | 0.066 |
| AllSNP _R2 | 99.80 | 43.53 | 500.99 | 45.16 | 600.79 | 31.25 | 0.17 | 0.071 |
| AllSNP _R3 | 55.17 | 34.21 | 536.14 | 41.3 | 591.3 | 30.33 | 0.093 | 0.058 |
| AllSNP _R4 | 58.89 | 33.55 | 527.66 | 40.38 | 586.55 | 30.13 | 0.10 | 0.057 |
| AllSNP _R5 | 82.67 | 40.63 | 531.19 | 44.74 | 613.86 | 31.71 | 0.14 | 0.066 |
| average | 76.41 | 38.51 | 524.76 | 43.2 | 601.17 | 31.03 | 0.13 | 0.064 |

RF – Random Forests; GBM – Gradient Boosting Machine, XgBoost – Extreme Gradient Boosting; Even – evenly spaced along the genome; $h^2$ – heritability; $\sigma_e^2$ – residual variance. † The SNPs with positive variable importance values.

**Supplementary Table S4.** Five-fold cross-validation scheme - individual subset estimates of average accuracy of genomic prediction by different subsets of SNPs from Brahman cow population

| Marker | Accuracy |
|---|---|
| RF400_R1 | 0.416 |
| RF400_R2 | 0.321 |
| RF400_R3 | 0.427 |
| RF400_R4 | 0.350 |
| RF400_R5 | 0.252 |
| average | 0.353 |
| RF1000_R1 | 0.336 |
| RF1000_R2 | 0.278 |
| RF1000_R3 | 0.513 |
| RF1000_R4 | 0.408 |
| RF1000_R5 | 0.278 |
| average | 0.363 |
| RF3000_R1 | 0.366 |
| RF3000_R2 | 0.361 |
| RF3000_R3 | 0.679 |
| RF3000_R4 | 0.368 |
| RF3000_R5 | 0.290 |
| average | 0.413 |
| RF18453_R1 | 0.371 |
| RF18453_R2 | 0.332 |
| RF18453_R3 | 0.631 |
| RF18453_R4 | 0.473 |
| RF18453_R5 | 0.294 |
| average | 0.420 |
| GBM400_R1 | 0.345 |
| GBM400_R2 | 0.318 |
| GBM400_R3 | 0.599 |
| GBM400_R4 | 0.080 |
| GBM400_R5 | 0.475 |
| average | 0.363 |
| GBM1000_R1 | 0.388 |
| GBM1000_R2 | 0.390 |
| GBM1000_R3 | 0.609 |
| GBM1000_R4 | 0.241 |
| GBM1000_R5 | 0.479 |
| average | 0.421 |
| GBM3000_R1 | 0.441 |
| GBM3000_R2 | 0.358 |
| GBM3000_R3 | 0.526 |
| GBM3000_R4 | 0.533 |
| GBM3000_R5 | 0.448 |
| average | 0.461 |
| GBM16600_R1 | 0.361 |

| | |
|---|---|
| GBM16600_R2 | 0.321 |
| GBM16600_R3 | 0.567 |
| GBM16600_R4 | 0.487 |
| GBM16600_R5 | 0.346 |
| average | 0.416 |
| Xgb400_R1 | 0.099 |
| Xgb400_R2 | 0.196 |
| Xgb400_R3 | 0.280 |
| Xgb400_R4 | 0.286 |
| Xgb400_R5 | 0.154 |
| average | 0.203 |
| Xgb1000_R1 | 0.287 |
| Xgb1000_R2 | 0.106 |
| Xgb1000_R3 | 0.343 |
| Xgb1000_R4 | 0.305 |
| Xgb1000_R5 | 0.241 |
| average | 0.256 |
| Xgb3000_R1 | 0.166 |
| Xgb3000_R2 | 0.299 |
| Xgb3000_R3 | 0.506 |
| Xgb3000_R4 | 0.402 |
| Xgb3000_R5 | 0.269 |
| average | 0.328 |
| Xgb9122_R1 | 0.279 |
| Xgb9122_R2 | 0.341 |
| Xgb9122_R3 | 0.573 |
| Xgb9122_R4 | 0.513 |
| Xgb9122_R5 | 0.260 |
| average | 0.393 |
| Even400_R1 | 0.202 |
| Even400_R2 | 0.084 |
| Even400_R3 | 0.204 |
| Even400_R4 | 0.223 |
| Even400_R5 | 0.165 |
| average | 0.176 |
| Even1000_R1 | 0.012 |
| Even1000_R2 | 0.181 |
| Even1000_R3 | 0.366 |
| Even1000_R4 | 0.216 |
| Even1000_R5 | 0.299 |
| average | 0.215 |
| Even3000_R1 | 0.025 |
| Even3000_R2 | 0.308 |
| Even3000_R3 | 0.499 |
| Even3000_R4 | 0.177 |
| Even3000_R5 | 0.440 |
| average | 0.290 |
| AllSNP _R1 | 0.375 |
| AllSNP _R2 | 0.293 |

| | |
|---|---|
| AllSNP _R3 | 0.612 |
| AllSNP _R4 | 0.501 |
| AllSNP _R5 | 0.342 |
| average | 0.425 |
